# Plasma tau proteins for the diagnosis of mild cognitive impairment and Alzheimer's disease: A systematic review and meta-analysis

**DOI:** 10.3389/fnagi.2022.942629

**Published:** 2022-07-25

**Authors:** Leian Chen, Xiaoqian Niu, Yuye Wang, Shuang Lv, Xiao Zhou, Ziyuan Yang, Dantao Peng

**Affiliations:** ^1^Department of Neurology, China-Japan Friendship Hospital, Beijing, China; ^2^Chinese Academy of Medical Sciences & Peking Union Medical College, Beijing, China; ^3^Peking University China-Japan Friendship School of Clinical Medicine, Beijing, China

**Keywords:** Alzheimer's disease, biomarker, tau, meta-analysis, mild cognitive impairment (MCI)

## Abstract

**Objective:**

Detecting plasma tau biomarkers used to be impossible due to their low concentrations in blood samples. Currently, new high-sensitivity assays made it a reality. We performed a systematic review and meta-analysis in order to test the accuracy of plasma tau protein in diagnosing Alzheimer's disease (AD) or mild cognitive impairment (MCI).

**Methods:**

We searched PubMed, Cochrane, Embase and Web of Science databases, and conducted correlation subgroup analysis, sensitivity analysis and publication bias analysis using R Programming Language.

**Results:**

A total of 56 studies were included. Blood t-tau and p-tau levels increased from controls to MCI to AD patients, and showed significant changes in pairwise comparisons of AD, MCI and normal cognition. P-tau217 was more sensitive than p-tau181 and p-tau231 in different cognition periods. In addition, ultrasensitive analytical platforms, immunomagnetic reduction (IMR), increased the diagnostic value of tau proteins, especially the diagnostic value of t-tau.

**Conclusion:**

Both t-tau and p-tau are suitable AD blood biomarkers, and p-tau217 is more sensitive than other tau biomarkers to differentiate MCI and AD. Detection techniques also have an impact on biomarkers' results. New ultrasensitive analytical platforms of IMR increase the diagnostic value of both t-tau and p-tau biomarkers.

**Systematic review registration:**

https://www.crd.york.ac.uk/PROSPERO/, registration number: CRD42021264701.

## Introduction

Alzheimer's disease (AD) is the most common progressive neurodegenerative disease among the elderly population and is the leading cause of dementia worldwide (Garre-Olmo, 2018). It is estimated that the number of patients suffering from Alzheimer's disease worldwide will reach 135 million by the end of 2050 (Prince et al., 2013). Mild cognitive impairment (MCI) is a predementia stage on the continuum of cognitive decline (Langa and Levine, 2014). Identification of MCI is important for early diagnosis and intervention of dementia. However, it is still a considerable challenge for clinicians to identify AD or MCI at an early stage due to no effective method.

Amyloid-β (Aβ) accumulation and tau neurofibrillary tangles are two characteristic pathological features of AD (Quintas-Neves et al., 2019). In 2018, the National Institute on Aging and Alzheimer's Association (NIA-AA) further emphasized the role of biomarkers in the AD biological definition. A renewed diagnostic scheme is based on biomarker evidence of amyloid (“A”), tau (“T”), and neurodegeneration (“N”), in which “A” was represented by Aβ, “T” represented by p-tau and “N” represented by t-tau or neurofilament light chain (NFL) (Jack et al., 2018). Both cerebrospinal fluid (CSF) and plasma can reflect the pathological situation in patients' brains. PET amyloid imaging is the most direct method to measure amyloid deposition in the living brain, but it cannot be popularized due to the cost and complexity (Snyder et al., 2014). CSF is considered an ideal biomarker source because it is in direct contact with brain tissue and can directly reflect the pathological changes in brain tissue (Jack et al., 2018). Nevertheless, acquiring cerebrospinal fluid requires invasive lumbar puncture, making most patients reluctant to this test (Thijssen et al., 2020). This highlights the need for less invasive and cheaper techniques. The use of blood in AD diagnostic procedures is promising because it is less invasive and affordable and has already been used to diagnose other diseases widespreadly (Khan, 2016). Therefore, finding blood AD biomarkers is a primary concern for doctors.

In recent years, there have been many studies on blood Aβ, and its diagnostic value for AD has been gradually recognized (Nakamura et al., 2018). However, individuals who are negative for Aβ biomarkers but positive for tau or neurodegenerative biomarkers are common in the real world (Hampel et al., 2021). Tau pathology is another main pathological feature of AD. Tau protein is most abundantly expressed in axons of central nervous system neurons. The most important role of tau protein is to promote assembly and stability of microtubules (Šimić et al., 2016). Total tau (t-tau) is composed of both phosphorylated and non-phosphorylated proteins. Phosphorylation is one of the most common post-translational modifications, changing the shape of tau molecule and regulating biological activity. Most of the phosphorylation sites are on Ser-Pro and Thr-Pro motives. Hyperphosphorylated tau proteins aggregate into neurofibrillary tangles (NFTs), which play a critical role in AD pathological processes. Hyperphosphorylated tau is a precipitant of neurodegeneration and cognitive and functional decline in AD (Blennow and Zetterberg, 2018). It is necessary to combine tau when diagnosing AD. Blood test for tau-related proteins was not available easily in the past. With the advance of several ultra-high sensitive technologies, detecting blood total tau and phosphorylated tau becomes reliable (Fossati et al., 2019). There are many studies on blood tau, but the outcomes appear inconsistent (Teunissen et al., 2022). The varied results reduce the clinical value. Therefore, we performed a meta-analysis of plasma tau proteins to distinguish AD and MCI cases from the normal controls, or MCI cases from AD patients at an early stage. We also compared the performance of different platforms, including traditional methods and next-generation analytical techniques (Zu and Bard, 2000; Hong et al., 2006; Rissin et al., 2010; Shinohara et al., 2021).

## Methods

This protocol was registered in PROSPERO (registration number: CRD42021264701) and conducted according to the Preferred Reporting Items for Systematic Reviews and Meta-analysis (PRISMA) checklist (Page et al., 2021).

### Search strategy

We searched PubMed, Cochrane, Embase and Web of Science from inception to June 26, 2021. The complete search strategy was ((((“Alzheimer Disease”[Mesh]) OR (Alzheimer^*^)) OR (AD[Title/Abstract])) AND ((((“tau Proteins”[Mesh]) OR (tau)) OR (t-tau)) OR (p-tau))) AND (((blood) OR (plasma)) OR (serum)). A manual search of references of all retrieved studies, relevant reviews and systematic reviews was also carried out for further supplement.

### Selection criteria

**Figure 1:**
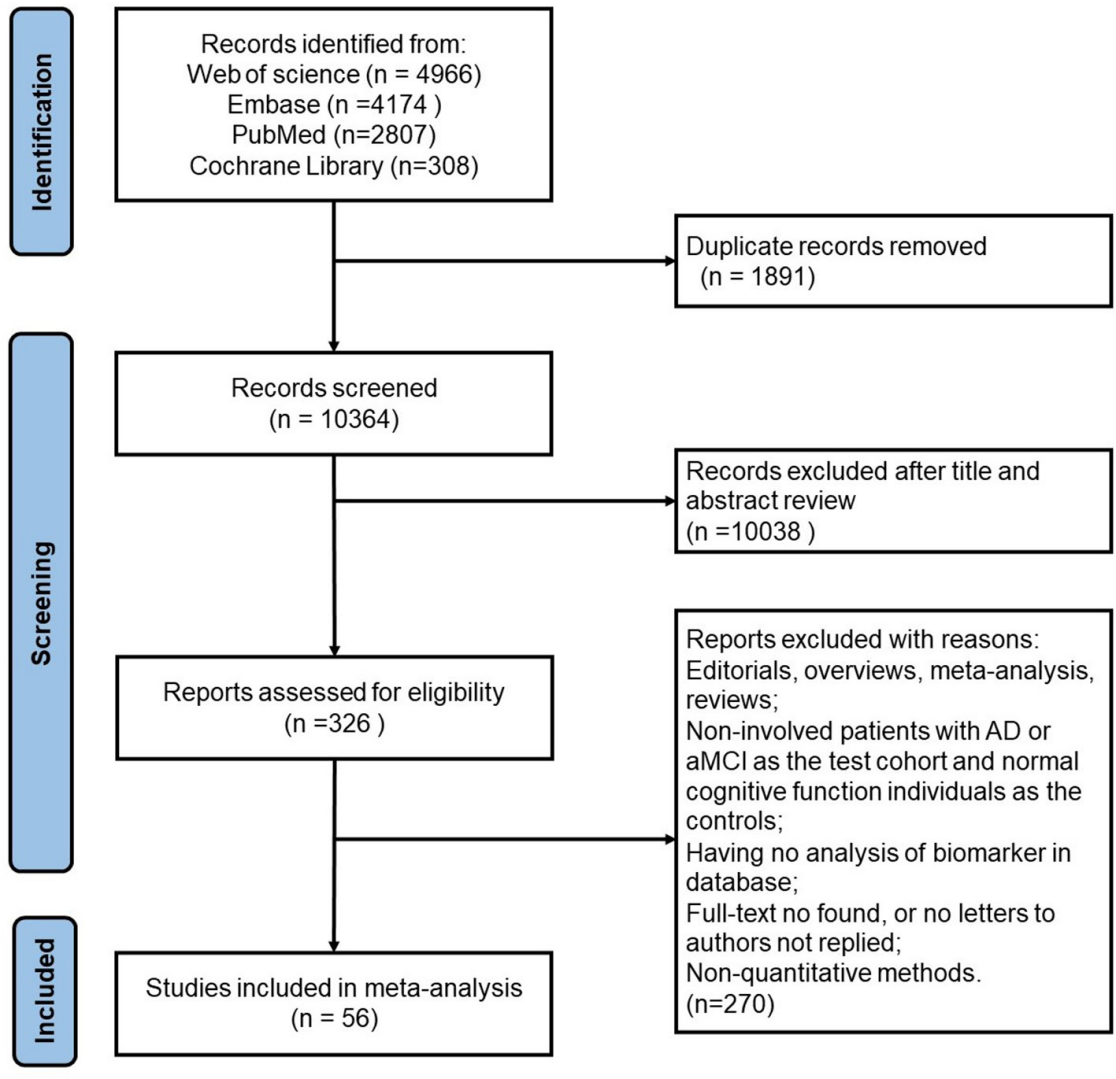
Study selection flowchart.

Studies that met the following criteria were included: (1) patients with AD or MCI were selected as the test cohort, and cognitively normal individuals as the controls; (2) those include cross-sectional, case-control or cohort studies; (3) the Mini-Mental State Examination (MMSE), cerebrospinal fluid biomarkers, NIAA-AA guidelines, PET or MRI were used as reference standards; (4) corresponding data of blood biomarkers (t-tau, p-tau181, p-tau217) was available. We excluded duplicate publications, reviews, meta-analyses, abstracts, animal and cell studies, editorials, case reports, and letters. Figure 1 illustrates the study selection procedure.

### Data extraction

Data were extracted independently by two authors who participated in data extraction training (Chen and Niu), including study characteristics (article number, publication year, first author, study design, country or region, sample size, age, relevant subgroups and detection methods) and outcomes (MMSE score, blood biomarker types and values). Data were obtained from cross-sectional studies and baseline measurements from cohort studies with clinical follow-up. Finally, all the input data were checked by the third author. Biomarkers and MMSE were expressed as Mean [Standard Deviation (SD)], Median [IQR (Interquartile Range)] or Median (Range) concentration, and ultimately expressed as Mean (SD) through the calculation methods (Luo et al., 2018; McGrath et al., 2020). If only standard error (SE) was reported, we used a conversion formula (*SD* = $\sqrt{N\cdot SE}$) to calculate SD.

### Quality assessment of studies

**Figure 2:**
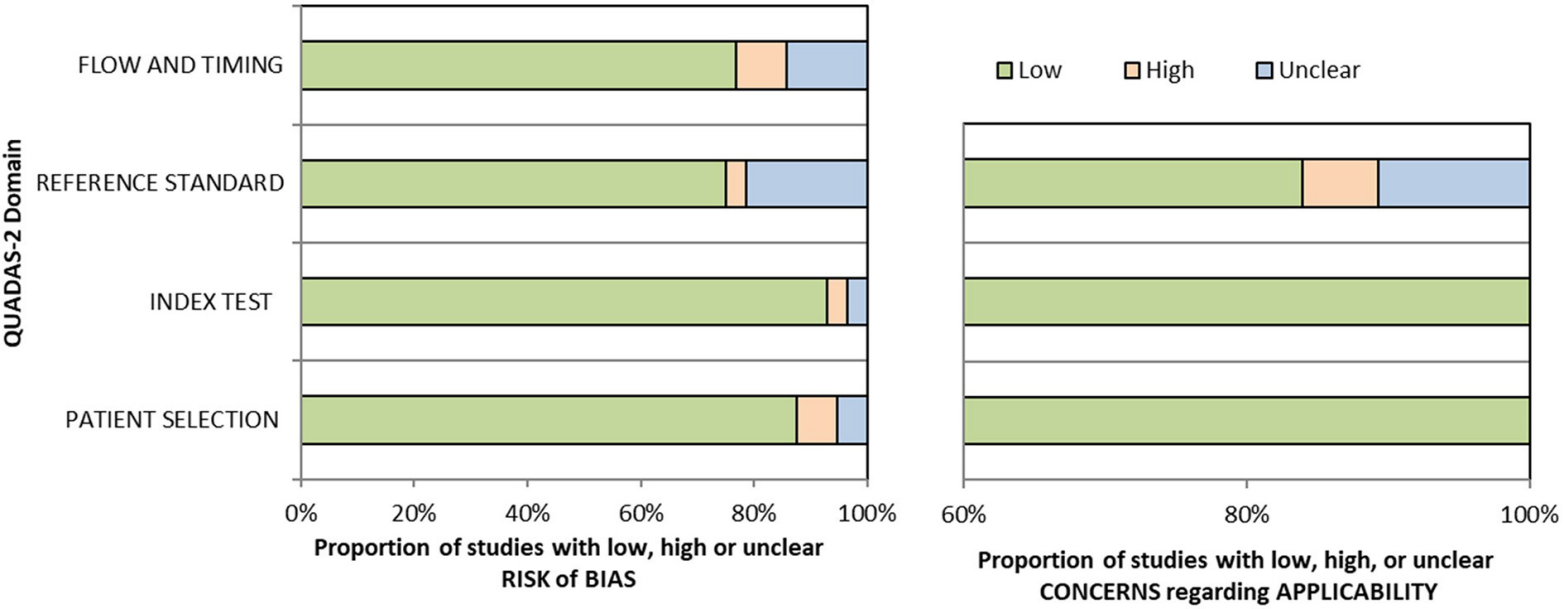
Methodologic quality assessment of eligible studies by using Quality Assessment of Diagnostic Accuracy Studies-2 (QUADAS-2) checklist.

Quality was assessed using the Quality Assessment of Diagnostic Accuracy Studies-2 (QUADAS-2) tool (Whiting et al., 2011). The tool contains four domains: patient selection, index test, reference standard, flow and timing. Each domain was assessed based on the risk of bias, and the four domains were also assessed on suitability. Clinical applicability was generally classified into three levels: “low,” “high” or “unclear.” A field is considered to exhibit a low risk of bias if all questions related to it mostly answer “yes.” However, if the answer to one or more signaling questions is “no,” the field is considered to exhibit a high risk of bias. Figure 2 depicts the methodological quality assessment results.

### Statistical analysis

Random effects meta-analysis was used in all analyses. There were huge variabilities in biomarker concentrations between assay platforms in different laboratories worldwide. Thus we utilized the ratio of means (ROM) between two groups as the pooled effect measure for each study. According to previous studies, the ratio of means (ROM) method is equal to mean difference (MD) and standardized mean difference (SMD) method for pooling continuous outcomes in meta-analysis (Friedrich et al., 2011). A ROM above 1 means the biomarker concentration of patients is higher, while a ROM below 1 implies the biomarker concentration is lower in patients. The publication bias test was assessed to evaluate whether the combined effect value was affected by the positive results of some studies using Egger's test and funnel plot. Sensitivity analysis was used to assess the stability and reliability of meta-analysis combined results, as well as whether the combined results changed significantly under the influence of individual studies. Meta-regression was used to identify the source of heterogeneity and then subgroup analysis was used to reduce heterogeneity within each subgroup significantly. A *p*-value of 0.05 or less was considered significant. A 95% confidence interval (CI) without one was also considered significant. All statistical analyses were performed using R version 4.1.2.

## Results

### Study selection and description

Through literature searching and screening, a total of 12,255 articles were found, and 10,364 were left after deduplication. By reviewing titles and abstracts, 10,038 articles were excluded. Finally, 56 articles were included in the systematic review and meta-analysis after full-text reading. If there were several separate studies in an article, we treated them as independent studies. Supplementary Tables 1, 2 show the characteristics of the included studies. All studies were based on plasma biomarkers. Among all the included studies, there were 45 studies on normal controls (NC) and MCI, 40 studies on NC and AD, and 33 studies on MCI and AD. We conducted subgroup analysis according to tau protein types and the analysis methods. The forest plots, funnel plots and sensitivity analyses are shown in Supplementary Figures 1–21.

### Discrimination of MCI and control groups

**Figure 3:**
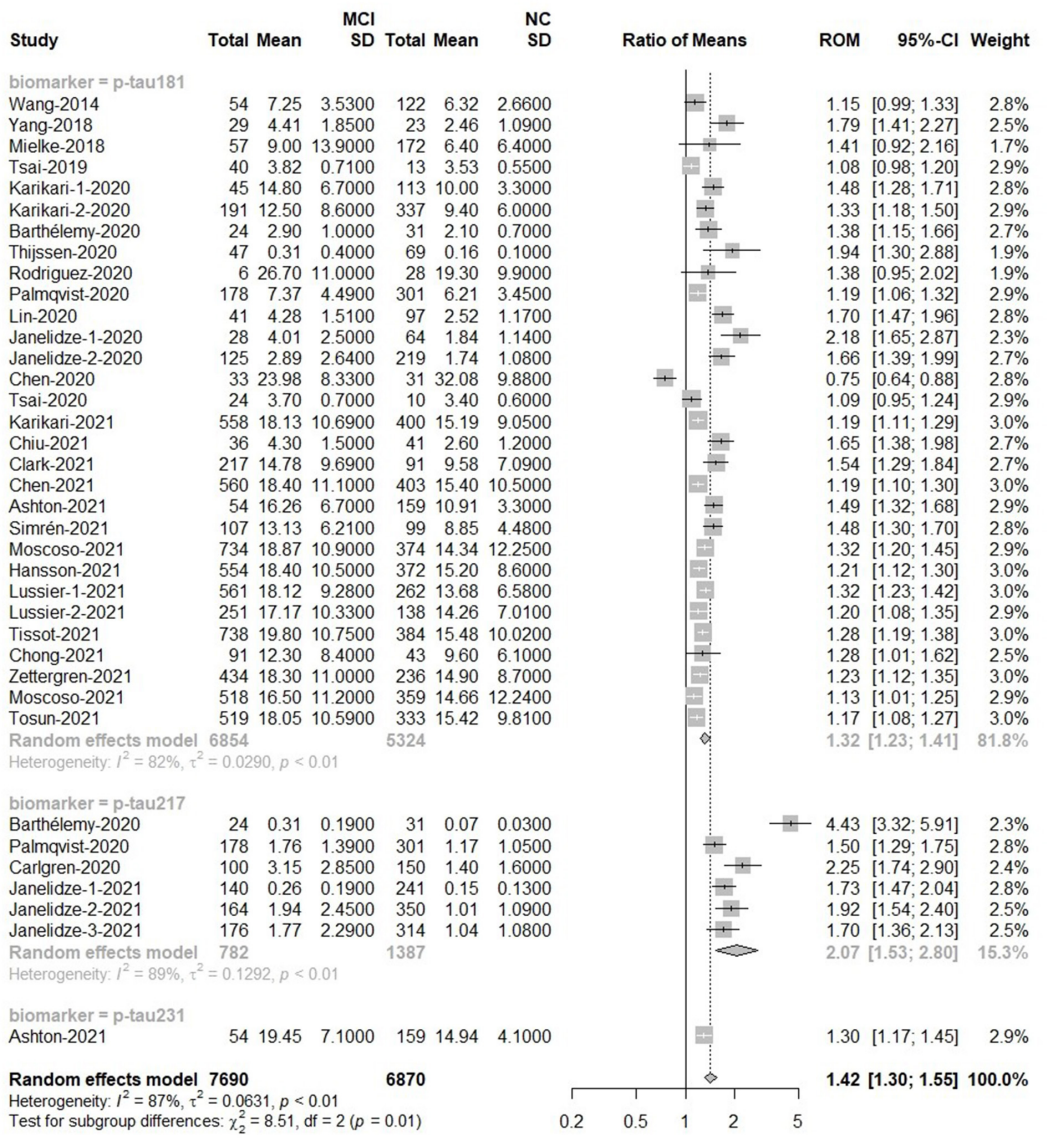
MCI to control ratio for blood p-tau: Forest plot of the ROMs (with 95%CI) for each tau subtype between MCI and normal cognitive controls. A ROM above one means that p-tau proteins were elevated in the MCI groups. Total, total sample size; Mean, mean value; SD, standard deviation; ROM, ratio of means; CI, confidence interval; MCI, mild cognitive impairment; NC, normal cognitive.

Among MCI (*n* = 7,690) subjects and NC (*n* = 6,870) subjects, there was a difference in overall p-tau [Ratio = 1.42, 95% CI = (1.30, 1.55), *p* < 0.0001]. P-tau181, p-tau217 and p-tau231 had high levels among MCI people than control groups. P-tau217 had better discriminative accuracy for MCI than p-tau181 and p-tau231. Forest plot for p-tau is displayed in Figure 3. In addition, there was no significant difference in the overall mean value of t-tau between the two groups [Ratio = 1.11, 95%CI = (0.97, 1.27), *p* = 0.12; Supplementary Figure 1].

Tau can be phosphorylated at multiple sites, but most assays only detect the phosphorylation of one specific aminoacid. To circumvent this variability in methods, we used ratios of means (ROM) for meta-analysis and combined the analyses of relevant forms of a specific protein into one. For the analysis methods in p-tau, these methods showed different detection capabilities. ELISA [Ratio = 0.93, 95%CI = (0.61, 1.41)] and Mass Spectrometry [MS; Ratio = 2.46, 95%CI = (0.78, 7.70)] could not detect the difference of p-tau protein between MCI groups and control groups. There were differences between MCI patients and control people when p-tau proteins were detected by IMR [Ratio = 1.41, 95% CI = (1.13, 1.77)], Meso Scale Discovery [MSD; Ratio = 1.71, 95%CI = (1.57, 1.86)] or single-molecule array [Simoa; Ratio = 1.28, 95%CI = (1.23, 1.33)]. As to Elecsys, the plasma p-tau level was much higher in MCI than that in control groups [Ratio = 2.25, 95% CI = (1.74, 2.90)), but only one study used Elecsys (Supplementary Figure 2).

For the t-tau biomarker, the overall results showed t-tau concentration in MCI was higher than that in normal people [Ratio = 1.36, 95%CI = (1.12, 1.63)]. The results showed no significant difference in the ELISA, Simoa, MS and MSD subgroups. Only IMR could detect this difference [Ratio = 2.20, 95%CI = (1.58, 3.06); Supplementary Figure 3].

### Discrimination of AD and control groups

**Figure 4:**
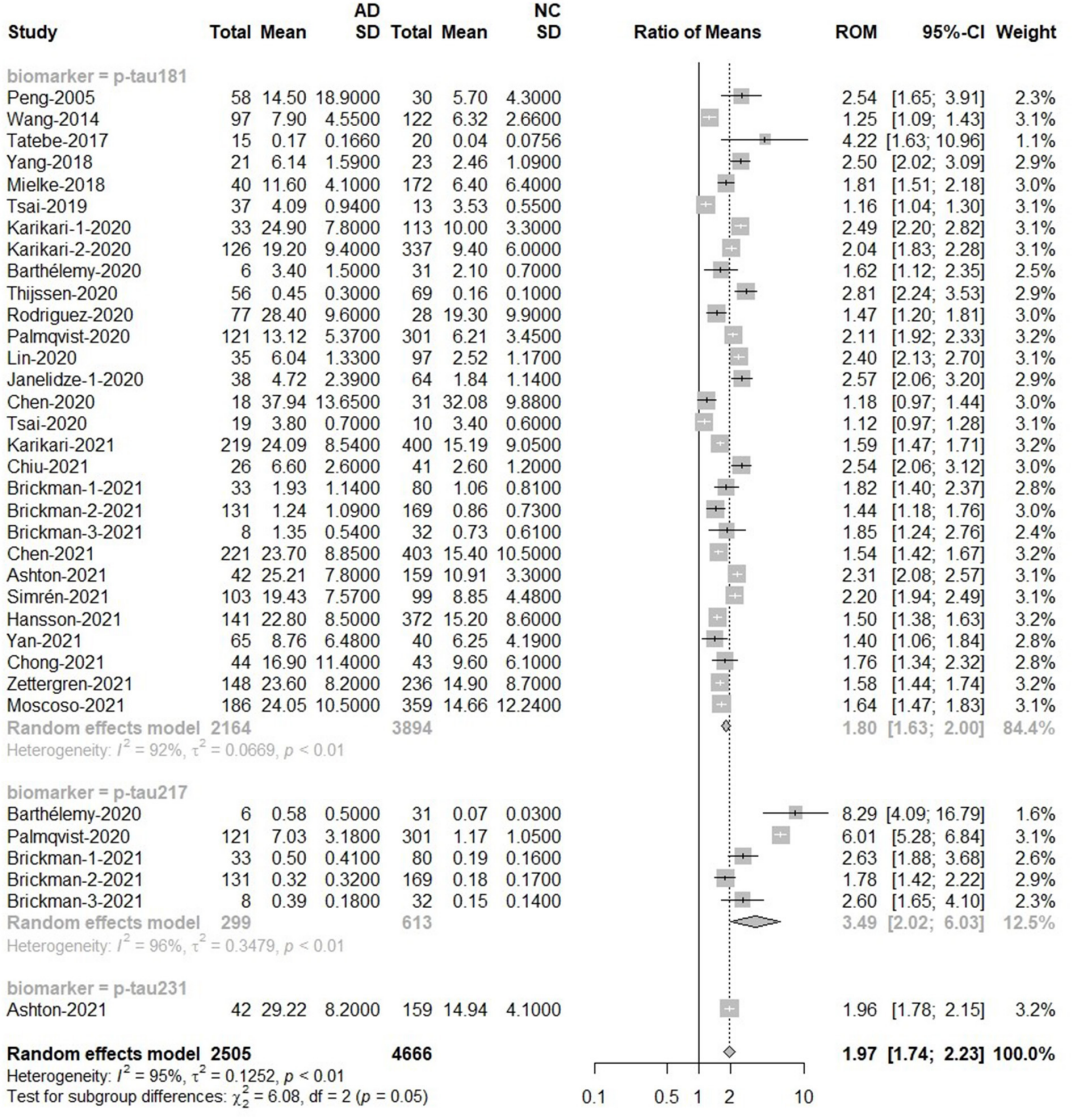
AD to control ratio for blood p-tau: Forest plot of the ROMs (with 95%CI) for each tau subtype between AD and normal cognitive controls. A ROM above one means that p-tau proteins were elevated in the AD groups. Total, total sample size; Mean, mean value; SD, standard deviation; ROM, ratio of means; CI, confidence interval; AD, Alzheimer's disease; NC, normal cognitive.

For p-tau, the overall results were based on the total number of 2,505 subjects in the AD group and 4,666 subjects in the control group. The p-tau value of the AD group was about twice that of the normal group [Ratio = 1.97, 95%CI (1.74–2.23), *p* < 0.0001]. P-tau181 [Ratio = 1.80, 95%CI = (1.63, 2.0)], p-tau217 [Ratio = 3.49, 95%CI = (2.02, 6.03)] and p-tau231 [Ratio = 1.96, 95%CI = (1.78, 2.15)] increased among AD groups. In addition, the ROM of p-tau217 was 3.49, revealing that plasma p-tau217 provided a better marker for AD detection than p-tau181 and p-tau231. The forest plot for p-tau is displayed in Figure 4. In addition, there was a significant difference in the mean value of t-tau between AD and control subjects [Ratio = 1.35, 95%CI = (1.12, 1.63), *p* = 0.0020; Supplementary Figure 4].

The next meta-analysis for methods showed the impact of measurement technique on blood p-tau testing results. The ELISA results showed that the concentration of p-tau protein in AD groups was 1.44 times higher than that in normal groups. When using Simoa or IMR, the p-tau concentration in AD groups was up to 1.8 times higher than that in normal groups. When the analysis was limited to MSD study, the value of p-tau in AD was higher [Ratio = 2.31, 95%CI = (1.82, 2.94)]. There was no significant difference between the two MS-based studies, although the levels of p-tau in AD were significantly higher [Ratio = 3.56, 95%CI = (0.72, 17.61); Supplementary Figure 5].

For the t-tau methods, AD subjects (*n* = 1,949) had higher t-tau levels than NC subjects (*n* = 3,203) [Ratio = 1.35, 95%CI = (1.12, 1.63)]. In studies using IMR, t-tau levels were significantly higher in AD patients [Ratio = 2.2, 95%CI = (1.58, 3.06)], and no significant difference was found in other subgroups [ELISA: Ratio = 0.84, 95%CI = (0.59, 1.21); Simoa: Ratio = 1.12, 95%CI = (1.00, 1.26); MS: Ratio = 1.15, 95%CI = (0.88, 1.52); Supplementary Figure 6].

### Discrimination of MCI and AD groups

**Figure 5:**
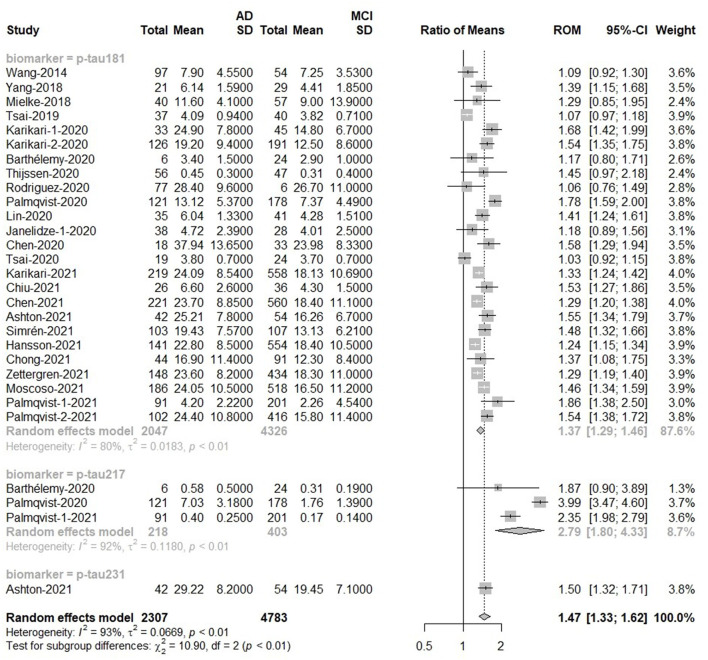
AD to MCI ratio for blood p-tau: Forest plot of the ROMs (with 95%CI) for each tau subtype between AD and MCI. A ROM above one means that p-tau proteins were elevated in the AD groups. Total, total sample size; Mean, mean value; SD, standard deviation; ROM, ratio of means; CI, confidence interval; AD, Alzheimer's disease; MCI, mild cognitive impairment.

There was a difference in overall p-tau between MCI (*n* = 4,783) and AD (*n* = 2,307) subjects. The meta-analyses results indicated that blood p-tau proteins had higher levels in AD patients compared with MCI [Ratio = 1.47, 95%CI = (1.33, 1.62), *p* < 0.0001]. The ROM of p-tau217 (Ratio = 2.79) was significantly higher than that of p-tau181 or p-tau 231 (Figure 5). In addition, t-tau also discriminated AD and MCI because t-tau was higher in AD patients than in MCI patients [Ratio = 1.24, 95%CI = (1.12, 1.38), *p* = 0.0020; Supplementary Figure 7].

No significant difference was observed in ELISA [Ratio = 1.31, 95%CI = (0.91, 1.88)], MSD [Ratio = 1.75, 95%CI = (0.98, 3.14)] and MS [Ratio = 1.33, 95% CI = (0.89, 1.99)]. Studies using IMR [Ratio = 1.26, 95%CI = (1.07, 1.47)] and Simoa [Ratio = 1.49, 95%CI = (1.38, 1.62)] had differences, and their average values were similar (Supplementary Figure 8).

For the t-tau analysis methods, AD subjects (*n* = 1,586) had higher t-tau levels than NC subjects [*n* = 1,832; Ratio = 1.24, 95%CI = (1.12, 1.38)]. There were significant differences in the following studies [MSD: Ratio = 1.23, 95%CI = (1.12, 1.35); Simoa: Ratio = 1.24, 95%CI = (1.05, 1.47); IMR: Ratio = 1.30, 95%CI = (1.15, 1.48)]. But on the whole, there is little difference between the average values of the two detection methods (Supplementary Figure 9).

## Discussion

This meta-analysis shows that Alzheimer's disease and MCI are associated with increased blood levels of t-tau and p-tau. In addition, IMR increases the diagnostic value of tau protein. These results are consistent with previous similar studies (Olsson et al., 2016; Lue et al., 2017; Koychev et al., 2021). To the best of our knowledge, we are the first to perform a meta-analysis on various types of tau proteins in a pairwise comparison way among normal people, MCI patients, and AD patients. A recent meta-analysis study has shown a higher specificity of p-tau217 in the diagnosis of AD (Qu et al., 2021). However, that study only compared the blood p-tau217 level between AD and MCI, excluding normal cognitive people. Our meta-analysis found blood p-tau217 level was increased from controls to MCI to AD patients, and p-tau217 was more sensitive than p-tau181 and p-tau231 in different cognition periods. This is because p-tau 217 is more tightly related to the formation of Aβ plaques in the brain (Janelidze et al., 2020). P-tau217 and p-tau181 reflect amyloid and tau protein deposition in the brain. Compared with plasma p-tau181, plasma p-tau217 correlates with the density of tau tangles and is also a better indicator of longitudinal tau accumulation. Plasma p-tau217 has been elevated in the early disease process of sporadic AD and autosomal dominant AD, so p-tau217 can better identify preclinical AD. Furthermore, in subjects with normal baseline tau PET scans, high levels of plasma p-tau217 were associated with increased tau PET in the entorhinal cortex, whereas this association was only present in cases with Aβ deposition, suggesting that plasma p-tau217 levels may be more closely related to Aβ deposition (Palmqvist et al., 2020; Pereira et al., 2021). Therefore, p-tau217 is more conducive to monitoring disease development.

Previous meta-analyses have shown that the new detection technology IMR can increase the diagnostic value of tau protein (Lue et al., 2017). Different tau proteins represent different pathological processes. For example, hyperphosphorylated tau proteins aggregate into neurofibrillary tangles (NFTs), a kind of AD pathological process associated with synapses loss, impairment of axonal transport, mitochondrial and cytoskeletal dysfunction (Gao et al., 2018). T-tau proteins are more correlated with ongoing axonal injury or degeneration, which in turn may indicate disease intensity (Zetterberg, 2017). Based on this, our study combined all tau protein biomarkers (p-tau181, p-tau217, p-tau231, and t-tau). The results showed that IMR could increase the diagnostic value of t-tau and p-tau for MCI and AD. Only IMR could detect the difference of t-tau in NC and MCI, NC and AD. Some researchers don't consider blood t-tau as an ideal biomarker of AD (Mattsson et al., 2016), mainly because of the short half-life in plasma and low specificity across Alzheimer's disease (AD) spectrum (Zetterberg, 2017). Our findings suggest that IMR is of great value in detecting t-tau differences, especially in normal cognitive people and MCI, normal cognitive people and AD. We also found that the ELISA results usually did not distinguish between normal and abnormal. One reason is that tau levels in the circulation are much lower than those in the CSF. Blood-brain barrier (BBB) acts as a selective filter, so that these brain proteins may be cleaved, modified or degraded before or after passing BBB (Kulichikhin et al., 2021). Biomarker levels in peripheral blood tend to be near the lower limits of detection of current ELISA assays and close to the low-end of the linear range of a calibration curve. Under these conditions, the ELISA assays lose their sensitivity for detecting narrow differences between biological samples (Lue et al., 2017). New approaches and technologies with superior sensitivity and specificity have also been applied to AD core biomarker analysis in biofluids, including IMR, Simoa, MSD and Elecsys immunoassays. The IMR plasma tau assay range is 0.002–2,500 pg/ml, while the range of Simoa plasma tau assay is 0–360 pg/ml (Lue et al., 2017), with much higher sensitivity than traditional ELISA (Kuhle et al., 2016). Simoa losses of tau protein molecules when purifying tau proteins, so the plasma tau levels detected with Simoa would be lower than that of IMR (Yang et al., 2017). That is why IMR could raise diagnostic value and perform better than other methods like ELISA and Simoa. With the help of IMR, the degree of nerve cell damage reflected by blood t-tau is promised to be an available indicator for judging the severity of the disease. And t-tau combined with p-tau is expected to provide more diagnostic information.

When the results are generalized into larger populations, other variables should be taken into consideration carefully. Previous studies suggested plasma p-tau levels can be affected by other diseases or factors. Multiple comorbidities, including a history of chronic kidney disease (CKD), hypertension, myocardial infarction (MI) and stroke, were found to be associated with elevated plasma p-tau181 and p-tau217 levels (Mielke et al., 2022). These confounding factors may influence physiological processes. For example, CKD reduces clearance of proteins in the blood, leading to the high p-tau and t-tau levels (Mielke et al., 2022). A history of stroke implies neuronal damage. There is a high incidence of these diseases in older people, and people at high risk for AD overlap with those with high risk for these diseases. Alzheimer's disease shares the same risk factors with CKD, hypertension, MI and stroke. Therefore, more relevant studies are needed to further study the changes of AD markers in chronic disease populations, and set different standards for chronic disease people. The blood AD biomarker results in chronic disease patients should also be interpreted with caution. The elevated tau biomarkers may indicate an early stage of AD pathology, or simply interference with plasma protein metabolism as a result of the disease state.

There are several limitations to this meta-analysis. First of all, certain eligible articles might not be included despite systemic reports. Second, there is large unexplained heterogeneity in these analyses, and thus the results need to be interpreted with caution. This heterogeneity could be due to a wide range of causes. Besides biological nature and demographic features of the participants, the heterogeneity also comes from either pre-analytical, analytical, or post-analytical procedures, such as assay sensitivity, platform, sample processing, storage condition, and test kit (Toledo et al., 2011; Babić et al., 2013). Standardizations of pre-analytical and analytical procedures in the collection, treatment, and storage of samples are also crucial because differences in sample handling can drastically influence results. Finally, our study only focused on single-form biomarkers. Further studies are needed to identify the discrimination of the combination or the ratio of tau and other biomarkers.

## Conclusion

Our meta-analysis demonstrated remarkable concentration alterations of diverse p-tau forms in the peripheral levels in pairwise comparisons of AD, MCI and normal cognition. This meta-analysis shows that t-tau and p-tau increase from controls to MCI to AD patients. P-tau217 is more sensitive than p-tau181 and p-tau231 in different cognition periods. In addition, new ultrasensitive analytical platforms, IMR, increases the diagnostic value of tau proteins, especially the diagnostic value of t-tau. Further research is required to validate blood tau levels among patients with disease states to make the AD blood biomarkers well accepted in clinical settings.

## Data availability statement

The original contributions presented in the study are included in the article/supplementary material, further inquiries can be directed to the corresponding author.

## Author contributions

LC, XN, and DP conceived and designed the study. LC and XN collected and analyzed data and wrote the original draft. YW, SL, and XZ provided the technical support. DP and ZY revised and finalized the manuscript. All authors contributed to the article and approved the submitted version.

## Funding

This work was supported by the National Natural Science Foundation of China (Grant No. 81974220) and Central health research project (Grant No. 2020ZD10).

## Conflict of interest

The authors declare that the research was conducted in the absence of any commercial or financial relationships that could be construed as a potential conflict of interest.

## Publisher's note

All claims expressed in this article are solely those of the authors and do not necessarily represent those of their affiliated organizations, or those of the publisher, the editors and the reviewers. Any product that may be evaluated in this article, or claim that may be made by its manufacturer, is not guaranteed or endorsed by the publisher.
