# Supplementary material for: Plasma tau proteins for the diagnosis of mild cognitive impairment and Alzheimer's disease: A systematic review and meta-analysis
Source: Front Aging Neurosci. 2022 Jul 25;14:942629. doi: 10.3389/fnagi.2022.942629 (PMC9358685; doi:10.3389/fnagi.2022.942629)

Supplementary Material

[1 Supplementary Table 1. Included studies. 2](#_Toc108479450)

[2 Supplementary Table 2. Characteristics of included studies. 11](#_Toc108479451)

[3 Supplementary Table 3. The modified QUADAS 18](#_Toc108479452)

[4 Supplementary Table 4. Quality assessment of cohort studies 19](#_Toc108479453)

[5 Supplementary Fig.1. MCI to control ratio for blood t-tau 24](#_Toc108479454)

[6 Supplementary Fig.2. Subgroup Analysis:MCI to control ratio for blood p-tau 25](#_Toc108479455)

[7 Supplementary Fig.3. Subgroup Analysis:MCI to control ratio for blood t-tau 26](#_Toc108479456)

[8 Supplementary Fig.4. AD to control ratio for blood t-tau 27](#_Toc108479457)

[9 Supplementary Fig.5. Subgroup Analysis: AD to control ratio for blood p-tau 28](#_Toc108479458)

[10 Supplementary Fig.6. Subgroup Analysis:AD to control ratio for blood t-tau 29](#_Toc108479459)

[11 Supplementary Fig.7. AD to MCI ratio for blood t-tau 30](#_Toc108479460)

[12 Supplementary Fig.8. Subgroup Analysis:AD to MCI ratio for blood p-tau 31](#_Toc108479461)

[13 Supplementary Fig.9. Subgroup Analysis:AD to MCI ratio for blood t-tau 32](#_Toc108479462)

[14 Supplementary Fig.10. Funnel plot of blood p-tau in MCI samples vs controls 33](#_Toc108479463)

[15 Supplementary Fig.11. Funnel plot of blood t-tau in MCI samples vs controls 34](#_Toc108479464)

[16 Supplementary Fig.12. Funnel plot of blood p-tau in AD samples vs controls 35](#_Toc108479465)

[17 Supplementary Fig.13. Funnel plot of blood t-tau in AD samples vs controls 36](#_Toc108479466)

[18 Supplementary Fig.14. Funnel plot of blood p-tau in AD samples vs MCI 37](#_Toc108479467)

[19 Supplementary Fig.15. Funnel plot of blood t-tau in AD samples vs MCI 38](#_Toc108479468)

[20 Supplementary Fig.16. Sensitivity analysis of blood p-tau in MCI samples vs controls 39](#_Toc108479469)

[21 Supplementary Fig.17. Sensitivity analysis of blood t-tau in MCI samples vs controls 40](#_Toc108479470)

[22 Supplementary Fig.18. Sensitivity analysis of blood p-tau in AD samples vs controls 41](#_Toc108479471)

[23 Supplementary Fig.19. Sensitivity analysis of blood t-tau in AD samples vs controls 42](#_Toc108479472)

[24 Supplementary Fig.20. Sensitivity analysis of blood p-tau in AD samples vs MCI 43](#_Toc108479473)

[25 Supplementary Fig.21. Sensitivity analysis of blood t-tau in AD samples vs MCI 44](#_Toc108479474)

# Supplementary Table 1. Included studies.

| No. | Title | Author+year | References |
| --- | --- | --- | --- |
| 1 | Evaluation of the role of plasma level of p-tau(181p) protein for clinical diagnosis of Alzheimer's disease | Peng 2005 | 1 |
| 2 | Tau is reduced in AD plasma and validation of employed ELISA methods | Sparks 2012 | 2 |
| 3 | Plasma tau levels in Alzheimer's disease | Zetterberg 2013 | 3 |
| 4 | Evaluation of Selenium, Redox Status and Their Association with Plasma Amyloid/Tau in Alzheimer's Disease | Krishnan 2014 | 4 |
| 5 | The efficacy of plasma biomarkers in early diagnosis of Alzheimer's disease | Wang 2014 | 5 |
| 6 | Plasma tau as a window to the brain-negative associations with brain volume and memory function in mild cognitive impairment and early Alzheimer's disease | Chiu 2014 | 6 |
| 7 | Plasma Aβ but not tau is related to brain PiB retention in early Alzheimer's disease | Tzen 2014 | 7 |
| 8 | Identifying biomarkers of dementia prevalent among amnestic mild cognitively impaired ethnic female patients | Grewal 2016 | 8 |
| 9 | Plasma tau in Alzheimer disease | Mattsson 2016 | 9 |
| 10 | Study on analysis of peripheral biomarkers for Alzheimer's disease diagnosis | Rani  2017 | 10 |
| 11 | Plasma levels of Aβ42 and Tau identified probable Alzheimer's dementia: Findings in two cohorts | Lue 2017 | 11 |
| 12 | Analytical performance of reagent for assaying tau protein in human plasma and feasibility study screening neurodegenerative diseases | Yang 2017 | 12 |
| 13 | Association of plasma total tau level with cognitive decline and risk of mild cognitive impairment or dementia in the Mayo Clinic study on aging | Mielke 2017 | 13 |
| 14 | Quantification of plasma phosphorylated tau to use as a biomarker for brain Alzheimer pathology: Pilot case-control studies including patients with Alzheimer's | Tatebe 2017 | 14 |
| 15 | Assay of Plasma Phosphorylated Tau Protein (Threonine 181) and Total Tau Protein in Early-Stage Alzheimer's Disease | Yang 2018 | 15 |
| 16 | Plasma phospho-tau181 increases with Alzheimer's disease clinical severity and is associated with tau- and amyloid-positron emission tomography | Mielke 2018 | 16 |
| 17 | Brain Amyloid-beta Deposition and Blood Biomarkers in Patients with Clinically Diagnosed Alzheimer's Disease | Li 2019 | 17 |
| 18 | Potential Value of Plasma Amyloid-beta, Total Tau, and Neurofilament Light for Identification of Early Alzheimer's Disease | Shi 2019 | 18 |
| 19 | Associations between Plasma Biomarkers and Cognition in Patients with Alzheimer's Disease and Amnestic Mild Cognitive Impairment: A Cross-Sectional and Longitudinal Study | Tsai 2019 | 19 |
| 20 | Plasma tau complements CSF tau and P-tau in the diagnosis of Alzheimer's disease | Fossati 2019 | 20 |
| 21 | Discriminative Accuracy of Plasma Phospho-tau217 for Alzheimer Disease vs Other Neurodegenerative Disorders | Palmqvist 2020 | 21 |
| 22 | Blood phosphorylated tau 181 as a biomarker for Alzheimer's disease: a diagnostic performance and prediction modelling study using data from four prospective cohorts | Karikari 2020 | 22 |
| 23 | Blood plasma phosphorylated-tau isoforms track CNS change in Alzheimer's disease | Barthélemy 2020 | 23 |
| 24 | Diagnostic value of plasma phosphorylated tau181 in Alzheimer's disease and frontotemporal lobar degeneration | Thijssen 2020 | 24 |
| 25 | The Validation of Multifactor Model of Plasma A beta(42) and Total-Tau in Combination With MoCA for Diagnosing Probable Alzheimer Disease | Jiao 2020 | 25 |
| 26 | Plasma p-tau181 accurately predicts Alzheimer's disease pathology at least 8 years prior to post-mortem and improves the clinical characterisation of cognitive decline | Rodriguez 2020 | 26 |
| 27 | Classifications of Neurodegenerative Disorders Using a Multiplex Blood Biomarkers-Based Machine Learning Model | Lin 2020 | 27 |
| 28 | A longitudinal examination of plasma neurofilament light and total tau for the clinical detection and monitoring of Alzheimer's disease | Sugarman 2020 | 28 |
| 29 | Longitudinal plasma p-tau217 is increased in early stages of Alzheimer's disease | Carlgren 2020 | 29 |
| 30* | Plasma P-tau181 in Alzheimer's disease: relationship to other biomarkers, differential diagnosis, neuropathology and longitudinal progression to Alzheimer's dementia* | Janelidze 2020* | 30 |
| 31 | Changes in Plasma Amyloid and Tau in a Longitudinal Study of Normal Aging, Mild Cognitive Imparment, and Alzheimer's Disease | Chen 2020 | 31 |
| 32 | The Correlations Between Plasma Fibrinogen With Amyloid-Beta and Tau Levels in Patients With Alzheimer's Disease | Fan 2020 | 32 |
| 33 | Indicators of rapid cognitive decline in amnestic mild cognitive impairment: The role of plasma biomarkers using magnetically labeled immunoassays | [Tsai 2020](https://pubmed.ncbi.nlm.nih.gov/?term=Tsai+CL&cauthor_id=32592947) | 33 |
| 34 | Plasma Tau and Neurofilament Light in Frontotemporal Lobar Degeneration and Alzheimer Disease | [Gala 2021](https://pubmed.ncbi.nlm.nih.gov/?term=Ill%C3%A1n-Gala+I&cauthor_id=33199433) | 34 |
| 35 | Diagnostic performance and prediction of clinical progression of plasma phospho-tau181 in the Alzheimer's Disease Neuroimaging Initiative | [Karikari 2021](https://pubmed-ncbi-nlm-nih-gov-443.webvpn.bjmu.edu.cn/?sort=date&term=Karikari+TK&cauthor_id=33106600) | 35 |
| 36 | Synergistic Association between Plasma Aβ(1-42) and p-tau in Alzheimer's Disease but Not in Parkinson's Disease or Frontotemporal Dementia | Chiu 2021 | 36 |
| 37 | Plasma neurofilament light and phosphorylated tau 181 as biomarkers of Alzheimer's disease pathology and clinical disease progression | [Clark 2021](https://www-ncbi-nlm-nih-gov-443.webvpn.bjmu.edu.cn/pubmed/?term=Clark%20C%5bAuthor%5d&cauthor=true&cauthor_uid=33766131) | 37 |
| 38 | Plasma Levels of Amyloid-β Peptides and Tau Protein in Mexican Patients with Alzheimer's Disease | [Mendieta 2021](https://pubmed-ncbi-nlm-nih-gov-443.webvpn.bjmu.edu.cn/?sort=date&term=Castillo-Mendieta+T&cauthor_id=34151786) | 38 |
| 39 | Plasma p-tau181, p-tau217, and other blood-based Alzheimer's disease biomarkers in a multi-ethnic, community study | [Brickman 2021](https://pubmed-ncbi-nlm-nih-gov-443.webvpn.bjmu.edu.cn/?sort=date&term=Brickman+AM&cauthor_id=33580742) | 39 |
| 40 | Prediction of future Alzheimer's disease dementia using plasma phospho-tau combined with other accessible measures | [Palmqvist 2021](https://pubmed-ncbi-nlm-nih-gov-443.webvpn.bjmu.edu.cn/?sort=date&term=Palmqvist+S&cauthor_id=34031605) | 40 |
| 41 | Longitudinal plasma phosphorylated tau 181 tracks disease progression in Alzheimer's disease | Chen 2021 | 41 |
| 42 | Detecting amyloid positivity in early Alzheimer's disease using combinations of plasma Aβ42/Aβ40 and p-tau | Janelidze 2021-1 | 42 |
| 43 | Associations of Plasma Phospho-Tau217 Levels With Tau Positron Emission Tomography in Early Alzheimer Disease | Janelidze 2021-2 | 43 |
| 44 | Plasma Biomarkers of Alzheimer's Disease in African Americans | Deniz 2021 | 44 |
| 45 | Plasma p-tau231: a new biomarker for incipient Alzheimer's disease pathology | Ashton 2021 | 45 |
| 46 | Blood biomarkers for dementia in Hispanic and non-Hispanic White adults | Gonzales 2021 | 46 |
| 47 | The diagnostic and prognostic capabilities of plasma biomarkers in Alzheimer's disease | Simrén2021 | 47 |
| 48 | Longitudinal Associations of Blood Phosphorylated Tau181 and Neurofilament Light Chain With Neurodegeneration in Alzheimer Disease | Moscoso 2021-1 | 48 |
| 49 | Plasma phosphorylated tau181 and neurodegeneration in Alzheimer's disease | Hansson 2021 | 49 |
| 50 | Plasma levels of phosphorylated tau 181 are associated with cerebral metabolic dysfunction in cognitively impaired and amyloid-positive individuals | Lussier 2021 | 50 |
| 51 | Plasma pTau181 predicts cortical brain atrophy in aging and Alzheimer’s disease | Tissot 2021 | 51 |
| 52 | The value of alzheimer's disease-associated neurofilament protein combined with AB1-42/P-TAU-181 ratio for the diagnosis of alzheimer's | Yan 2021 | 52 |
| 53 | Plasma P-tau181 to A beta 42 ratio is associated with brain amyloid burden and hippocampal atrophy in an Asian cohort of Alzheimer's disease patients with concomitant cerebrovascular disease | Chong 2021 | 53 |
| 54 | Association between polygenic risk score of Alzheimer's disease and plasma phosphorylated tau in individuals from the Alzheimer's Disease Neuroimaging Initiative | Zettergren 2021 | 54 |
| 55 | Time course of phosphorylated-tau181 in blood across the Alzheimer's disease spectrum | Moscoso 2021-2 | 55 |
| 56 | Detection of beta-amyloid positivity in Alzheimer's Disease Neuroimaging Initiative participants with demographics, cognition, MRI and plasma biomarkers | Tosun 2021 | 56 |

*There are two separate studies in this paper, so we treat them as two different studies.

**Reference:**

1. Peng DT, Xian-Hao XU, Cai XJJCJoG. Evaluation of the role of plasma level of p-tau(~ (181)p) protein for clinical diagnosis of Alzheimer′s disease. 2005.

2. Sparks DL, Kryscio RJ, Sabbagh MN, et al. Tau is reduced in AD plasma and validation of employed ELISA methods. *American journal of neurodegenerative disease.* 2012;1(1):99-106.

3. Zetterberg H, Wilson D, Andreasson U, et al. Plasma tau levels in Alzheimer's disease. *Alzheimer's research & therapy.* 2013;5(2):9.

4. Krishnan S, Rani P. Evaluation of selenium, redox status and their association with plasma amyloid/tau in Alzheimer's disease. *Biological trace element research.* 2014;158(2):158-165.

5. Wang T, Xiao S, Liu Y, et al. The efficacy of plasma biomarkers in early diagnosis of Alzheimer's disease. *International journal of geriatric psychiatry.* 2014;29(7):713-719.

6. Chiu MJ, Chen YF, Chen TF, et al. Plasma tau as a window to the brain-negative associations with brain volume and memory function in mild cognitive impairment and early Alzheimer's disease. *Human brain mapping.* 2014;35(7):3132-3142.

7. Tzen KY, Yang SY, Chen TF, et al. Plasma Aβ but not tau is related to brain PiB retention in early Alzheimer's disease. *ACS chemical neuroscience.* 2014;5(9):830-836.

8. Grewal R, Haghighi M, Huang S, et al. Identifying biomarkers of dementia prevalent among amnestic mild cognitively impaired ethnic female patients. *Alzheimer's research & therapy.* 2016;8(1):43.

9. Mattsson N, Zetterberg H, Janelidze S, et al. Plasma tau in Alzheimer disease. *Neurology.* 2016;87(17):1827-1835.

10. Rani P, Krishnan S, Rani Cathrine C. Study on Analysis of Periphe.ral Biomarkers for Alzheimer's Disease Diagnosis. *Frontiers in neurology.* 2017;8:328.

11. Lue LF, Sabbagh MN, Chiu MJ, et al. Plasma Levels of Aβ42 and Tau Identified Probable Alzheimer's Dementia: Findings in Two Cohorts. *Frontiers in aging neuroscience.* 2017;9:226.

12. Yang SY, Chiu MJ, Chen TF, et al. Analytical performance of reagent for assaying tau protein in human plasma and feasibility study screening neurodegenerative diseases. *Scientific reports.* 2017;7(1):9304.

13. Mielke MM, Hagen CE, Wennberg AMV, et al. Association of Plasma Total Tau Level With Cognitive Decline and Risk of Mild Cognitive Impairment or Dementia in the Mayo Clinic Study on Aging. *JAMA neurology.* 2017;74(9):1073-1080.

14. Tatebe H, Kasai T, Ohmichi T, et al. Quantification of plasma phosphorylated tau to use as a biomarker for brain Alzheimer pathology: pilot case-control studies including patients with Alzheimer's disease and down syndrome. *Molecular neurodegeneration.* 2017;12(1):63.

15. Yang CC, Chiu MJ, Chen TF, Chang HL, Liu BH, Yang SY. Assay of Plasma Phosphorylated Tau Protein (Threonine 181) and Total Tau Protein in Early-Stage Alzheimer's Disease. *Journal of Alzheimer's disease : JAD.* 2018;61(4):1323-1332.

16. Mielke MM, Hagen CE, Xu J, et al. Plasma phospho-tau181 increases with Alzheimer's disease clinical severity and is associated with tau- and amyloid-positron emission tomography. *Alzheimer's & dementia : the journal of the Alzheimer's Association.* 2018;14(8):989-997.

17. Li WW, Shen YY, Tian DY, et al. Brain Amyloid-β Deposition and Blood Biomarkers in Patients with Clinically Diagnosed Alzheimer's Disease. *Journal of Alzheimer's disease : JAD.* 2019;69(1):169-178.

18. Shi Y, Lu X, Zhang L, et al. Potential Value of Plasma Amyloid-β, Total Tau, and Neurofilament Light for Identification of Early Alzheimer's Disease. *ACS chemical neuroscience.* 2019;10(8):3479-3485.

19. Tsai CL, Liang CS, Lee JT, et al. Associations between Plasma Biomarkers and Cognition in Patients with Alzheimer's Disease and Amnestic Mild Cognitive Impairment: A Cross-Sectional and Longitudinal Study. *Journal of clinical medicine.* 2019;8(11).

20. Fossati S, Ramos Cejudo J, Debure L, et al. Plasma tau complements CSF tau and P-tau in the diagnosis of Alzheimer's disease. *Alzheimer's & dementia (Amsterdam, Netherlands).* 2019;11:483-492.

21. Palmqvist S, Janelidze S, Quiroz YT, et al. Discriminative Accuracy of Plasma Phospho-tau217 for Alzheimer Disease vs Other Neurodegenerative Disorders. *Jama.* 2020;324(8):772-781.

22. Karikari TK, Pascoal TA, Ashton NJ, et al. Blood phosphorylated tau 181 as a biomarker for Alzheimer's disease: a diagnostic performance and prediction modelling study using data from four prospective cohorts. *The Lancet Neurology.* 2020;19(5):422-433.

23. Barthélemy NR, Horie K, Sato C, Bateman RJ. Blood plasma phosphorylated-tau isoforms track CNS change in Alzheimer's disease. *The Journal of experimental medicine.* 2020;217(11).

24. Thijssen EH, La Joie R, Wolf A, et al. Diagnostic value of plasma phosphorylated tau181 in Alzheimer's disease and frontotemporal lobar degeneration. *Nature medicine.* 2020;26(3):387-397.

25. Jiao F, Yi F, Wang Y, et al. The Validation of Multifactor Model of Plasma Aβ (42) and Total-Tau in Combination With MoCA for Diagnosing Probable Alzheimer Disease. *Frontiers in aging neuroscience.* 2020;12:212.

26. Lantero Rodriguez J, Karikari TK, Suárez-Calvet M, et al. Plasma p-tau181 accurately predicts Alzheimer's disease pathology at least 8 years prior to post-mortem and improves the clinical characterisation of cognitive decline. *Acta neuropathologica.* 2020;140(3):267-278.

27. Lin CH, Chiu SI, Chen TF, Jang JR, Chiu MJ. Classifications of Neurodegenerative Disorders Using a Multiplex Blood Biomarkers-Based Machine Learning Model. *International journal of molecular sciences.* 2020;21(18).

28. Sugarman MA, Zetterberg H, Blennow K, et al. A longitudinal examination of plasma neurofilament light and total tau for the clinical detection and monitoring of Alzheimer's disease. *Neurobiology of aging.* 2020;94:60-70.

29. Mattsson-Carlgren N, Janelidze S, Palmqvist S, et al. Longitudinal plasma p-tau217 is increased in early stages of Alzheimer's disease. *Brain : a journal of neurology.* 2020;143(11):3234-3241.

30. Janelidze S, Mattsson N, Palmqvist S, et al. Plasma P-tau181 in Alzheimer's disease: relationship to other biomarkers, differential diagnosis, neuropathology and longitudinal progression to Alzheimer's dementia. Nature medicine. 2020;26(3):379-386.

31. Chen TB, Lai YH, Ke TL, et al. Changes in Plasma Amyloid and Tau in a Longitudinal Study of Normal Aging, Mild Cognitive Impairment, and Alzheimer's Disease. *Dementia and geriatric cognitive disorders.* 2019;48(3-4):180-195.

32. Fan DY, Sun HL, Sun PY, et al. The Correlations Between Plasma Fibrinogen With Amyloid-Beta and Tau Levels in Patients With Alzheimer's Disease. *Frontiers in neuroscience.* 2020;14:625844.

33. Tsai CL, Liang CS, Yang CP, et al. Indicators of rapid cognitive decline in amnestic mild cognitive impairment: The role of plasma biomarkers using magnetically labeled immunoassays. *Journal of psychiatric research.* 2020;129:66-72.

34. Illán-Gala I, Lleo A, Karydas A, et al. Plasma Tau and Neurofilament Light in Frontotemporal Lobar Degeneration and Alzheimer Disease. *Neurology.* 2021;96(5):e671-e683.

35. Karikari TK, Benedet AL, Ashton NJ, et al. Diagnostic performance and prediction of clinical progression of plasma phospho-tau181 in the Alzheimer's Disease Neuroimaging Initiative. *Molecular psychiatry.* 2021;26(2):429-442.

36. Chiu MJ, Yang SY, Chen TF, Lin CH, Blennow KJACN. Synergistic Association between Plasma Aβ1-42 and p-tau in Alzheimer's Disease but Not in Parkinson's Disease or Frontotemporal Dementia. 2021.

37. Clark C, Lewczuk P, Kornhuber J, et al. Plasma neurofilament light and phosphorylated tau 181 as biomarkers of Alzheimer's disease pathology and clinical disease progression. *Alzheimer's research & therapy.* 2021;13(1):65.

38. Castillo-Mendieta T, Arana-Lechuga Y, Campos-Peña V, et al. Plasma Levels of Amyloid-β Peptides and Tau Protein in Mexican Patients with Alzheimer's Disease. *Journal of Alzheimer's disease : JAD.* 2021;82(s1):S271-s281.

39. Brickman AM, Manly JJ, Honig LS, et al. Plasma p-tau181, p-tau217, and other blood-based Alzheimer's disease biomarkers in a multi-ethnic, community study. *Alzheimer's & dementia : the journal of the Alzheimer's Association.* 2021;17(8):1353-1364.

40. Palmqvist S, Tideman P, Cullen N, et al. Prediction of future Alzheimer's disease dementia using plasma phospho-tau combined with other accessible measures. *Nature medicine.* 2021;27(6):1034-1042.

41. Chen SD, Huang YY, Shen XN, et al. Longitudinal plasma phosphorylated tau 181 tracks disease progression in Alzheimer's disease. *Translational psychiatry.* 2021;11(1):356.

42. Janelidze S, Palmqvist S, Leuzy A, et al. Detecting amyloid positivity in early Alzheimer's disease using combinations of plasma Aβ42/Aβ40 and p-tau. *Alzheimer's & dementia : the journal of the Alzheimer's Association.* 2022;18(2):283-293.

43. Janelidze S, Berron D, Smith R, Strandberg O, Hansson OJJn. Associations of Plasma Phospho-Tau217 Levels With Tau Positron Emission Tomography in Early Alzheimer Disease.78(2):149-156.

44. Deniz K, Ho C, Malphrus KG, Reddy JS, Ertekin-Taner NJJoAsdJ. Plasma Biomarkers of Alzheimer's Disease in African Americans. 2020;79(1):1-12.

45. Ashton NJ, Pascoal TA, Karikari TK, et al. Plasma p-tau231: a new biomarker for incipient Alzheimer's disease pathology. *Acta neuropathologica.* 2021;141(5):709-724.

46. Gonzales MM, Short MI, Satizabal CL, et al. Blood biomarkers for dementia in Hispanic and non-Hispanic White adults. *Alzheimer's & dementia (New York, N Y).* 2021;7(1):e12164.

47. Simrén J, Leuzy A, Karikari TK, et al. The diagnostic and prognostic capabilities of plasma biomarkers in Alzheimer's disease. *Alzheimer's & dementia : the journal of the Alzheimer's Association.* 2021;17(7):1145-1156.

48. Moscoso A, Grothe MJ, Ashton NJ, et al. Longitudinal Associations of Blood Phosphorylated Tau181 and Neurofilament Light Chain With Neurodegeneration in Alzheimer Disease. *JAMA neurology.* 2021;78(4):396-406.

49. Hansson O, Cullen N, Zetterberg H, Blennow K, Mattsson-Carlgren N. Plasma phosphorylated tau181 and neurodegeneration in Alzheimer's disease. *Annals of clinical and translational neurology.* 2021;8(1):259-265.

50. Lussier FZ, Benedet AL, Therriault J, et al. Plasma levels of phosphorylated tau 181 are associated with cerebral metabolic dysfunction in cognitively impaired and amyloid-positive individuals. *Brain communications.* 2021;3(2):fcab073.

51. Tissot C, A LB, Therriault J, et al. Plasma pTau181 predicts cortical brain atrophy in aging and Alzheimer's disease. *Alzheimer's research & therapy.* 2021;13(1):69.

52. Congyang Yan, Maojun Miao, Bing Liu, et al. The value of alzheimer's disease-associated neurofilament protein combined with AB1-42/P-TAU-181 ratio for the diagnosis of alzheimer's. *Acta Medica Mediterranea,.* 2021;37:1653-1657.

53. Chong JR, Ashton NJ, Karikari TK, et al. Plasma P-tau181 to Aβ42 ratio is associated with brain amyloid burden and hippocampal atrophy in an Asian cohort of Alzheimer's disease patients with concomitant cerebrovascular disease. *Alzheimer's & dementia : the journal of the Alzheimer's Association.* 2021;17(10):1649-1662.

54. Zettergren A, Lord J, Ashton NJ, et al. Association between polygenic risk score of Alzheimer's disease and plasma phosphorylated tau in individuals from the Alzheimer's Disease Neuroimaging Initiative. *Alzheimer's research & therapy.* 2021;13(1):17.

55. Moscoso A, Grothe MJ, Ashton NJ, et al. Time course of phosphorylated-tau181 in blood across the Alzheimer's disease spectrum. *Brain : a journal of neurology.* 2021;144(1):325-339.

56. Tosun D, Veitch D, Aisen P, et al. Detection of β-amyloid positivity in Alzheimer's Disease Neuroimaging Initiative participants with demographics, cognition, MRI and plasma biomarkers. *Brain communications.* 2021;3(2):fcab008.

# Supplementary Table 2. Characteristics of included studies.

| No. | Year | First author | Study design | Region | Biomarker | | Methods | Center | Sample size,n | | Cohort | Age, mean(SD),year |
| --- | --- | --- | --- | --- | --- | --- | --- | --- | --- | --- | --- | --- |
| 1 | 2005 | Peng | Cross-sectional | China | p-tau181 | | ELISA | NA | 88 | | NC/AD | 77.8(4.3)/77.2(3.9) |
| 2 | 2012 | Sparks | Prospective | USA | t-tau | | ELISA | NA | 206 | | NC/MCI/AD | 78.5(7.3)/78.7(12.3)/84.4(7.1) |
| 3 | 2013 | Zetterberg | Cross-sectional | Sweden | t-tau | | Simoa | NA | 154 | | NC/MCI/AD | 74 (6.7)/68 (9.3)/75 (6.2) |
| 4 | 2014 | Krishnan | Cross-sectional | India | t-tau | | ELISA | NA | 105 | | NC/AD/VD | 65.22(9.34)/71.00(8.72)/64.94(12.33) |
| 5 | 2014 | Wang | Cross-sectional | China | t-tau,  p-tau181 | | ELISA | NA | 273 | | NC/MCI/AD | 73.7(8.4)/76.6(9.1)/73.7(9.4) |
| 6 | 2014 | Chiu | Cross-sectional | Taiwan | t-tau | | IMR | NA | 60 | | NC/MCI/AD | 64.4(9.5)/71.2(9.7)/69.3(9.4) |
| 7 | 2014 | Tzen | Cross-sectional | Taiwan | t-tau | | IMR | NA | 45 | | NC/MCI/AD | 63.7(7.9)/69.2(10.4)/64.9(11.5) |
| 8 | 2016 | Grewal | Cross-sectional | USA | t-tau,  p-tau | | ELISA | NA | 75 | | NC/MCI | 72.97(1.60)/76.23(1.47); |
| 9 | 2016 | Mattsson | Prospective | USA,  Canada | t-tau | | Simoa | ADNI | 563 | | NC/MCI/AD | 75.9(4.9)/74.7(7.5)/75.2(7.4) |
| 10 | 2016 | Mattsson | Prospective | Sweden | t-tau | | Simoa | BioFINDER | 721 | | NC/SCD/MCI/AD | 72.9(4.9)/71.2(5.5)/76.4(4.7) |
| 11 | 2017 | Rani | Cross-sectional | India | t-tau | | ELISA | NA | 90 | | NC/AD | 67.46(8.29)/71.73 (8.84) |
| 12 | 2017 | Lue | Cross-sectional | Taiwan,  USA | t-tau | | IMR | BSHRI,NTUH | 124 | | NC/AD | 68.1(1.3)/75.9(1.4) |
| 13 | 2017 | Yang | Cross-sectional | Taiwan | t-tau | | IMR | NTUH | 215 | | NC/MCI/AD | 64.6 (8.6)/71.0 (10.3)/72.2 (9.9) |
| 14 | 2017 | Mielke | Prospective | USA | t-tau | | Simoa | MCSA | 458 | | NC/MCI | 80.8(4.8)/79.9(7.4) |
| 15 | 2017 | Tatebe | Cross-sectional | Japan | p-tau181 | | Simoa | NA | 35 | | NC/AD | 76.3(3.2)/77.4(7.7) |
| 16 | 2018 | Yang | Cross-sectional | Taiwan | p-tau181;  t-tau | | IMR | NA | 73 | | NC/MCI/AD | 67.5(7.1)/71.0(8.7)/78.8(7.9) |
| 17 | 2018 | Mielke | Prospective | USA | p-tau181;  t-tau | | Simoa,MSD | MCSA,ADRC | 269 | | NC/MCI/AD | 71.9(9.5)/71.4(10.7)/67.7(9.2) |
| 18 | 2019 | Li | Cross-sectional | China | t-tau | | Simoa | NA | 84 | | NC/MCI/AD | 61.78(10.52)/64.60(9.3)/ 68.39(9.65) |
| 19 | 2019 | Shi | Cross-sectional | China | t-tau | | Simoa | NA | 155 | | NC/MCI | 64.77(7.40)/64.53(7.68) |
| 20 | 2019 | Tsai | Cross-sectional | Taiwan | p-tau181;  t-tau | | IMR | NA | 90 | | NC/MCI/AD | 64.4(5.7)/72.4(7.6)/77.0(8.3) |
| 21 | 2019 | Fossati | Cross-sectional | USA | t-tau | Simoa | | NA | 97 | NC/AD | | 67.71 (8.54)/72.81 (9.69) |
| 22 | 2020 | Thomas K Karikari | NA | USA | p-tau181 | Simoa | | TRIAD,  BioFINDER | 989 | TRIAD:NC/MCI/AD BioFINDER-2：NC/MCI/AD | | TRIAD: 69.2 (9.7)/72.6 (6.8)/64.6 (9.2) BioFINDER-2:63.1 (5.0)/70.6 (8.1)/74.0 (6.9)/ |
| 23 | 2020 | Nicolas R Barthélemy | Cross-sectional | Washington | t-tau;  p-tau217;  p-tau181 | MS | | NA | 92 | NC/non aMCI/pre-AD/aMCI/AD | | 73(5)/75(8)/74(6)/76(6)/74(8) |
| 24 | 2020 | Elisabeth H Thijssen | Cross-sectional | NA | p-tau181 | MSD | | NA | 362 | NC/MCI/AD | | 60.6(22)/60.8(14)/65.0 (9) |
| 25 | 2020 | Jiao | Cross-sectional | China | t-tau | IMR | | NA | 97 | NC/AD | | 67.9(9.5)/68.1(9.0) |
| 26 | 2020 | Rodriguez | Prospective | Sweden | p-tau181 | Simoa | | Maudsley and King’s Healthcare Partners DCR | 111 | NC/MCI/AD | | 82.2(6.5)/87.1(6.1)/81.7(7.6) |
| 27 | 2020 | Palmqvist | Cross-sectional | Sweden | p-tau217;  p-tau181;  t-tau | MSD | | BioFINDER-2 | 699 | NC/MCI/AD | | 65.97(15.49)/70.08(8.99)/74.24(5.78) |
| 28 | 2020 | Lin | Cross-sectional | Taiwan | p-tau181;  t-tau | IMR | | NA | 291 | NC/MCI/AD | | 64.0(7.8)/72.9(7.9)/75.2(11.6) |
| 29 | 2020 | Sugarman | Prospective | USA | t-tau | Simoa | | BU ADC Clinical Core Registry | 579 | NC/MCI/AD | | 72.38(7.69)/74.99(7.24)/76.74(8.12) |
| 30 | 2020 | Carlgren | Prospective | Sweden | p-tau217 | MSD | | BioFINDER | 250 | NC/MCI | | 71.53(5.11)/70.24(5.54) |
| 31 | 2020 | Janelidze | Prospective | Sweden | p-tau181 | Simoa | | BioFINDER | 182 | NC/MCI/AD | | 74.74(5.86)/73.07(7.03)/72.64(8.47) |
| 32 | 2020 | Janelidze | Prospective | Sweden | p-tau181;  t-tau | Simoa | | BioFINDER | 344 | NC/MCI | | 71.63(4.95)/71.24(6.81) |
| 33 | 2020 | Chen | Prospective | Taiwan | p-tau181,  t-tau | ELISA | | NA | 82 | NC/MCI/AD | | 75.67(7.44)/78.84(5.77)/78.98(5.47) |
| 34 | 2020 | Fan | Cross-sectional | China | t-tau | Simoa | | NA | 135 | NC/MCI | | 68.42(8.52)/66.31(9.53) |
| 35 | 2020 | [Tsai](https://pubmed.ncbi.nlm.nih.gov/?term=Tsai+CL&cauthor_id=32592947) | Prospective | Taiwan | p-tau181;  t-tau | IMR | | NA | 53 | NC/MCI/AD | | 64.6(6.3)/72.8(7.7)/72.8(7.7) |
| 36 | 2021 | [Ignacio Illán-Gala](https://pubmed.ncbi.nlm.nih.gov/?term=Ill%C3%A1n-Gala+I&cauthor_id=33199433) | Cross-sectional | USA | t-tau | Simoa | | NA | 265 | AD/NC | | 65.2(10)/52.2(13) |
| 37 | 2021 | [Thomas K Karikari](https://pubmed-ncbi-nlm-nih-gov-443.webvpn.bjmu.edu.cn/?sort=date&term=Karikari+TK&cauthor_id=33106600) | Prospective | USA,  Canada | p-tau181 | Simoa | | ANDI | 1177 | NC/MCI/AD | | 74.19(6.58)/72.48（7.56）/74.3(8.09) |
| 38 | 2021 | Ming-Jang Chiu | Cross-sectional | Taiwan,  Sweden | p-tau181 | IMR | | NA | 158 | NC/MCI/AD | | 65.1(6.8)/72.7(7.8)/76.7(7.5) |
| 39 | 2021 | Christopher Clark | Cross-sectional | Switzerland | p-tau181 | Simoa | | NA | 218 | NC/MCI | | 68.53 ( 7.31)/74 (6.6) |
| 40 | 2021 | Tzayaka Castillo-Mendieta | Cross-sectional | Mexico | t-tau;  p-tau | ELISA | | NA | 101 | NC/MCI/AD | | 80.5(8.18)/82.08(8.62)/78.0(9.07) |
| 41 | 2021 | Adam M Brickman | Cross-sectional | USA,  Spain | p-tau181;  p-tau217 | MSD | | NA | 453 | NC/AD | | Pathological status:84.93 (7.45)/87.38 (6.04) Clinical status:81.01 (6.31)/82.99 (6.49) PET amyloid status:82.16 (5.19)/84.25 (4.55) |
| 42 | 2021 | Sebastian Palmqvist | Prospective | Sweden | p-tau181;  p-tau217 | Simoa | | BioFINDER,  ANDI | 883 | AD/MCI | | biofinder:72.1 (4.91)/70.2 (5.73) ANDI:73.2 (6.98)/71.2 (7.14) |
| 43 | 2021 | Shi-Dong Chen | Prospective | USA | p-tau;  t-tau | Simoa | | ADNI | 1184 | NC/MCI/AD | | 74.9(0.33)/72.9(0.34)/75.3(0.53) |
| 44 | 2021 | Shorena Janelidze-1 | Cross-sectional | Sweden | p-tau217 | MSD | | BioFINDER | 895 | NC/MCI | | BioFINDER1:72 (5.22)/71 (6.74) BioFINDER2:64 (16.38)/71 (7.48) |
| 45 | 2021 | Shorena Janelidze-2 | Prospective | Sweden | p-tau217 | MSD | | BioFINDER | 490 | NC/MCI | | 64.3(16.38) /72.1(7.77) |
| 46 | 2021 | Kaancan Deniz | Cross-sectional | USA | t-tau | Simoa | | NA | 321 | NC/AD | | 82.7(8.15)/78.2(8.97) |
| 47 | 2021 | Nicholas J Ashton | Prospective | Canada | p-tau181 | Simoa | | NA | 313 | NC/MCI/AD/non AD | | 69.2 (10.2)/69.8 (7.1)/65.7 (9.2)/66.7 (7.1) |
| 48 | 2021 | Mitzi M Gonzales | Cross-sectional | USA,  Spain | t-tau | Simoa | | NA | 1843 | NC/MCI/AD | | 63(8)/72(8)/70(9) 73(9)/75(8)/75(9) |
| 49 | 2021 | Joel Simrén | Prospective | Finland,  Italy,  England | p-tau181 | Simoa | | NA | 309 | NC/MCI/AD | | 73 (6.14)/74.47 (5.89)/76.35 |
| 50 | 2021 | Alexis Moscoso-1 | Prospective | Sweden | p-tau181 | Simoa | | ADNI | 1113 | NC/CI | | 74.8 (6.6)/73.6 (8) |
| 51 | 2021 | Oskar Hansson | Prospective | Sweden | p-tau181 | Simoa | | ADNI | 1067 | NC/MCI/AD | | 73.6(5.8)/71.9(7.4)/74.4(8.2) |
| 52 | 2021 | Firoza Z Lussier | Cross-sectional | Sweden | p-tau181 | Simoa | | ADNI | 1212 | Cross-sectional：NC/MCI Longitudinal:  NC/MCI | | Cross-sectional:73.00(7.48)/72.48(7.81) Longitudinal：75.12(7.83)/71.59(8.44) |
| 53 | 2021 | Cécile Tissot | Cross-sectional | NA | p-tau181 | Simoa | | ADNI | 1122 | NC/MCI | | 74.40 (6.50)/73.61 (7.94) |
| 54 | 2021 | Congy ang yan | Cross-sectional | China | p-tau181 | ELISA | | NA | 105 | NC/AD | | 66.46(4.23)/68.12(4.25) |
| 55 | 2021 | Joyce R. Chong | Cross-sectional | Singapore | p-tau181;  t-tau | Simoa | | NA | 200 | NC/MCI/AD/VAD | | 74(6)/76(6)/77(8)/75(9) |
| 56 | 2021 | Anna Zettergren | Cross-sectional | Sweden | p-tau181 | Simoa | | ADNI | 818 | NC/MCI/AD | | 73.8 (6.1)/72.6 (7.8)/75.5 (7.9) |
| 57 | 2021 | Alexis Moscoso-2 | Cross-sectional | USA,  Canada | p-tau181 | Simoa | | ADNI | 1063 | NC/MCI/AD | | 74.7 (6.7)/72.8 (7.9)/75.1 (7.8) |
| 58 | 2021 | Duygu Tosun | Cross-sectional | USA | p-tau181 | Simoa | | ADNI | 852 | NC/MCI | | 73.39(5.97)/72(7.45) |

# Supplementary Table 3. The modified QUADAS

| Patient Selection | 1-Was a consecutive or random sample of patients enrolled? Yes/No/Unclear 2-Was a case-control design avoided? Yes/No/Unclear 3-Did the study avoid inappropriate exclusions? Yes/No/Unclear 4-Could the selection of patients have introduced bias? RISK: LOW/HIGH/UNCLEAR 5-Are there Concerns That the Included Patients and Setting Do Not Match the Review Question? CONCERN: LOW/HIGH/UNCLEAR |
| --- | --- |
| Index Test | 6-Were the index test results interpreted without knowledge of the results of the reference standard? Yes/No/Unclear 7-If a threshold was used, was it prespecified? Yes/No/Unclear 8-Could the Conduct or Interpretation of the Index Test Have Introduced Bias? RISK: LOW/HIGH/UNCLEAR have introduced bias?  9-Is there concern that the index test, its conduct, or interpretation differ from the review question? CONCERN: LOW /HIGH/UNCLEAR |
| Reference Standard | 10-Is the reference standard likely to correctly classify the target condition? Yes/No/Unclear  11-Were the reference standard results interpreted without knowledge of the results of the index test? Yes/No/Unclear 12-Could the Reference Standard, Its Conduct, or Its Interpretation Have Introduced Bias? RISK: LOW /HIGH/UNCLEAR 13-Are there Concerns That the Target Condition as Defined by the Reference Standard Does Not Match the Question? CONCERN: LOW/HIGH/UNCLEAR |
| Flow and Timing | 14-Was there an appropriate interval between the index test and reference standard? Yes/No/Unclear 15-Did all patients receive the same reference standard? Yes/No/Unclear 16-Were all patients included in the analysis? Yes/No/Unclear 17-Could the Patient Flow Have Introduced Bias? RISK: LOW/HIGH/UNCLEAR |

# Supplementary Table 4. Quality assessment of cohort studies

| First author | Year | 1 | 2 | 3 | 4 | 5 | 6 | 7 | 8 | 9 | 10 | 11 | 12 | 13 | 14 | 15 | 16 | 17 |
| --- | --- | --- | --- | --- | --- | --- | --- | --- | --- | --- | --- | --- | --- | --- | --- | --- | --- | --- |
| Ignacio Illán-Gala | 2021 | Yes | No | Yes | Low | Low | Yes | Yes | Low | Low | Yes | Yes | Low | Low | No | Yes | Yes | Low |
| Thomas K Karikari | 2021 | Yes | Yes | Yes | Low | Low | Yes | Yes | Low | Low | Yes | Yes | Low | Low | No | Yes | Yes | Low |
| Ming-Jang Chiu | 2021 | No | No | Yes | Low | Low | Yes | No | Unclear | Low | Unclear | Unclear | Unclear | Low | No | No | Yes | High |
| Christopher Clark | 2021 | Yes | No | Yes | Low | Low | Yes | No | Low | Low | Yes | Yes | Low | Low | Unclear | Yes | Yes | Low |
| Tzayaka Castillo-Mendieta | 2021 | Yes | No | Yes | Low | Low | No | No | High | Low | Unclear | Unclear | Unclear | Low | Unclear | Unclear | Yes | Unclear |
| Adam M Brickman | 2021 | No | Yes | Yes | Low | Low | Yes | Yes | Low | Low | Yes | Yes | Low | Low | Yes | No | Yes | Low |
| Sebastian Palmqvist | 2021 | Yes | Yes | Yes | Low | Low | No | Yes | Unclear | Low | Yes | Yes | Low | Low | Yes | Yes | Yes | Low |
| Shi-Dong Chen | 2021 | Yes | Yes | Yes | Low | Low | Yes | Yes | Low | Low | Yes | Yes | Low | Low | Yes | No | Yes | Low |
| Shorena Janelidze-1 | 2021 | Yes | No | Yes | Low | Low | Yes | Yes | Low | Low | Yes | Yes | Low | Low | Yes | No | Yes | Low |
| Shorena Janelidze-2 | 2021 | Yes | Yes | Yes | Low | Low | Yes | Yes | Low | Low | Yes | Yes | Low | Low | Yes | Yes | Yes | Low |
| Kaancan Deniz | 2021 | Unclear | No | Yes | Unclear | Low | Yes | Yes | Low | Low | Yes | Yes | Low | Low | Yes | Yes | Yes | Low |
| Nicholas J Ashton | 2021 | Yes | Yes | Yes | Low | Low | Yes | Yes | Low | Low | Yes | Yes | Low | Low | Yes | Yes | Yes | Low |
| Mitzi M Gonzales | 2021 | No | Yes | Yes | Low | Low | Yes | Yes | Low | Low | Unclear | Unclear | Unclear | Low | Yes | No | No | High |
| Joel Simrén | 2021 | No | No | No | High | Low | Yes | Yes | Low | Low | Unclear | Yes | Unclear | Low | Unclear | Yes | Yes | Low |
| Alexis Moscoso -1 | 2021 | Yes | Yes | Yes | Low | Low | Yes | Yes | Low | Low | Yes | Yes | Low | Low | No | Yes | No | High |
| Oskar Hansson | 2021 | Yes | Yes | Yes | Low | Low | Yes | Yes | Low | Low | Yes | Yes | Low | Low | Yes | Yes | No | Low |
| Firoza Z Lussier | 2021 | Yes | Yes | Yes | Low | Low | Yes | Yes | Low | Low | Yes | Yes | Low | Low | Yes | Yes | Yes | Low |
| Cécile Tissot | 2021 | Yes | Yes | Yes | Low | Low | Yes | Yes | Low | Low | Unclear | Yes | Unclear | Low | Yes | No | No | High |
| Congy ang yan | 2021 | Yes | Yes | Yes | Low | Low | Yes | Yes | Low | Low | Yes | Yes | Low | Low | Yes | Yes | Yes | Low |
| Joyce R. Chong | 2021 | Yes | Yes | Yes | Low | Low | Yes | Yes | Low | Low | Yes | Yes | Low | Low | Yes | Yes | No | Low |
| Anna Zettergren | 2021 | Yes | Yes | Yes | Low | Low | Yes | Yes | Low | Low | Yes | Yes | Low | Low | Yes | Yes | Yes | Low |
| Alexis Moscoso-2 | 2021 | Yes | Yes | Yes | Low | Low | Yes | Yes | Low | Low | Yes | Yes | Low | Low | Yes | Yes | Yes | Low |
| Duygu Tosun | 2021 | Yes | Yes | Yes | Low | Low | Yes | Yes | Low | Low | Yes | Yes | Low | Low | Yes | Yes | No | Low |
| Thomas K Karikari | 2020 | Yes | Yes | Yes | Low | Low | Yes | Yes | Low | Low | Yes | Yes | Low | Low | Yes | Yes | Yes | Low |
| Shorena Janelidze | 2020 | Yes | Yes | Yes | Low | Low | Yes | Yes | Low | Low | Yes | Yes | Low | Low | Yes | Yes | Yes | Low |
| Nicolas R Barthélemy | 2020 | Yes | Yes | Yes | Low | Low | Yes | Yes | Low | Low | Yes | Yes | Low | Low | Yes | Yes | Yes | Low |
| Elisabeth H Thijssen | 2020 | Yes | Yes | Yes | Low | Low | Yes | Yes | Low | Low | Yes | Yes | Low | Low | Yes | Yes | Yes | Low |
| Fubin Jiao | 2020 | Yes | Yes | Yes | Low | Low | Yes | Yes | Low | Low | Yes | Yes | Low | Low | Yes | Yes | Yes | Low |
| Juan Lantero Rodriguez | 2020 | Yes | Yes | Yes | Low | Low | Yes | Yes | Low | Low | Yes | Yes | Low | Low | Yes | Yes | Yes | Low |
| Sebastian Palmqvist | 2020 | Yes | Yes | Yes | Low | Low | Yes | Yes | Low | Low | Yes | Yes | Low | Low | Yes | No | No | High |
| Chin-Hsien Lin | 2020 | Unclear | Yes | Yes | Unclear | Low | Yes | Yes | Low | Low | Unclear | Unclear | Unclear | Unclear | Unclear | Unclear | Yes | Unclear |
| Michael A Sugarman | 2020 | Yes | Yes | Yes | Low | Low | Yes | Yes | Low | Low | Yes | Yes | Low | Low | Yes | Yes | Yes | Low |
| Niklas MattssonCarlgren | 2020 | Unclear | Yes | Unclear | High | Low | Yes | Yes | Low | Low | Yes | Yes | Low | Low | Yes | Yes | Yes | Low |
| Ting-Bin Chen | 2020 | Unclear | Yes | Unclear | Unclear | Low | Yes | Yes | Low | Low | Yes | Yes | Low | Low | Yes | Yes | Yes | Low |
| Dong-Yu Fan | 2020 | Yes | Yes | No | High | Low | Yes | Yes | Low | Low | Yes | Yes | Low | Low | Unclear | Yes | Yes | Low |
| Chia-Lin Tsai | 2020 | Yes | Yes | Yes | Low | Low | Yes | Yes | Low | Low | Yes | Yes | Low | Low | Yes | Yes | Yes | Low |
| Wei-Wei Li | 2019 | Yes | Yes | Yes | Low | Low | Yes | Yes | Low | Low | Yes | Yes | Low | Low | Yes | Yes | Yes | Low |
| Yachen Shi | 2019 | Yes | Yes | Yes | Low | Low | Yes | Yes | Low | Low | Yes | Yes | Low | Low | Yes | Yes | Yes | Low |
| Chia-Lin Tsai | 2019 | Yes | Yes | Yes | Low | Low | Yes | Yes | Low | Low | Yes | Yes | Low | Low | Yes | Yes | Yes | Low |
| Silvia Fossati | 2019 | Unclear | Yes | Yes | Low | Low | Yes | Yes | Low | Low | Yes | Yes | Low | Low | Yes | Yes | Yes | Low |
| Che-Chuan Yang | 2018 | Yes | Yes | Yes | Low | Low | No | No | High | Low | No | No | High | High | Unclear | Yes | Yes | Unclear |
| Michelle M Mielke | 2018 | Yes | Yes | Yes | Low | Low | Yes | Yes | Low | Low | Yes | Yes | Low | Low | Yes | Yes | Yes | Low |
| Palaniswamy Rani | 2017 | Unclear | Yes | No | High | Low | Yes | Yes | Low | Low | Unclear | Yes | Unclear | Low | Yes | Yes | Yes | Low |
| Lih-Fen Lue | 2017 | Yes | Yes | Yes | Low | Low | Yes | Yes | Low | Low | Unclear | Unclear | Unclear | Unclear | Yes | No | Yes | Low |
| Shieh-Yueh Yang | 2017 | Yes | Yes | Yes | Low | Low | Yes | Yes | Low | Low | No | No | High | High | Unclear | Unclear | Yes | Unclear |
| Michelle M Mielke | 2017 | Yes | Yes | Yes | Low | Low | Yes | Yes | Low | Low | Yes | Yes | Low | Low | Yes | Yes | Yes | Low |
| Harutsugu Tatebe | 2017 | Unclear | Yes | Yes | Low | Low | Yes | Yes | Low | Low | Yes | Yes | Low | Low | Yes | Yes | Yes | Low |
| Rinko Grewal | 2016 | Yes | Yes | Yes | Low | Low | Yes | Yes | Low | Low | Unclear | Unclear | Unclear | Unclear | Unclear | Unclear | Yes | Unclear |
| Niklas Mattsson | 2016 | Yes | Yes | Yes | Low | Low | Yes | Yes | Low | Low | Yes | Yes | Low | Low | Yes | Yes | Yes | Low |
| Sreeram Krishnan | 2014 | Yes | Yes | No | Low | Low | Yes | Yes | Low | Low | Unclear | Unclear | Unclear | Unclear | Unclear | Unclear | Yes | Unclear |
| Tao Wang | 2014 | Yes | Yes | Yes | Low | Low | Yes | Yes | Low | Low | Unclear | Unclear | Unclear | Unclear | Unclear | Unclear | Yes | Unclear |
| Ming-Jang Chiu | 2014 | Yes | Yes | Yes | Low | Low | Yes | Yes | Low | Low | Yes | Yes | Low | Low | Yes | Yes | Yes | Low |
| Kai-Yuan Tzen | 2014 | Yes | Yes | Yes | Low | Low | Yes | Yes | Low | Low | Yes | Yes | Low | Low | Yes | Yes | Yes | Low |
| Henrik Zetterberg | 2013 | Yes | Yes | Yes | Low | Low | Yes | Yes | Low | Low | Yes | Yes | Low | Low | Yes | Yes | Yes | Low |
| D Larry Sparks | 2012 | Yes | Yes | Yes | Low | Low | Yes | Yes | Low | Low | No | Yes | Low | High | Yes | Yes | Yes | Low |
| Dantao Peng | 2005 | No | Yes | Yes | Low | Low | Yes | Yes | Low | Low | Unclear | Unclear | Unclear | Unclear | Unclear | Unclear | Yes | Unclear |

# Supplementary Fig.1. MCI to control ratio for blood t-tau


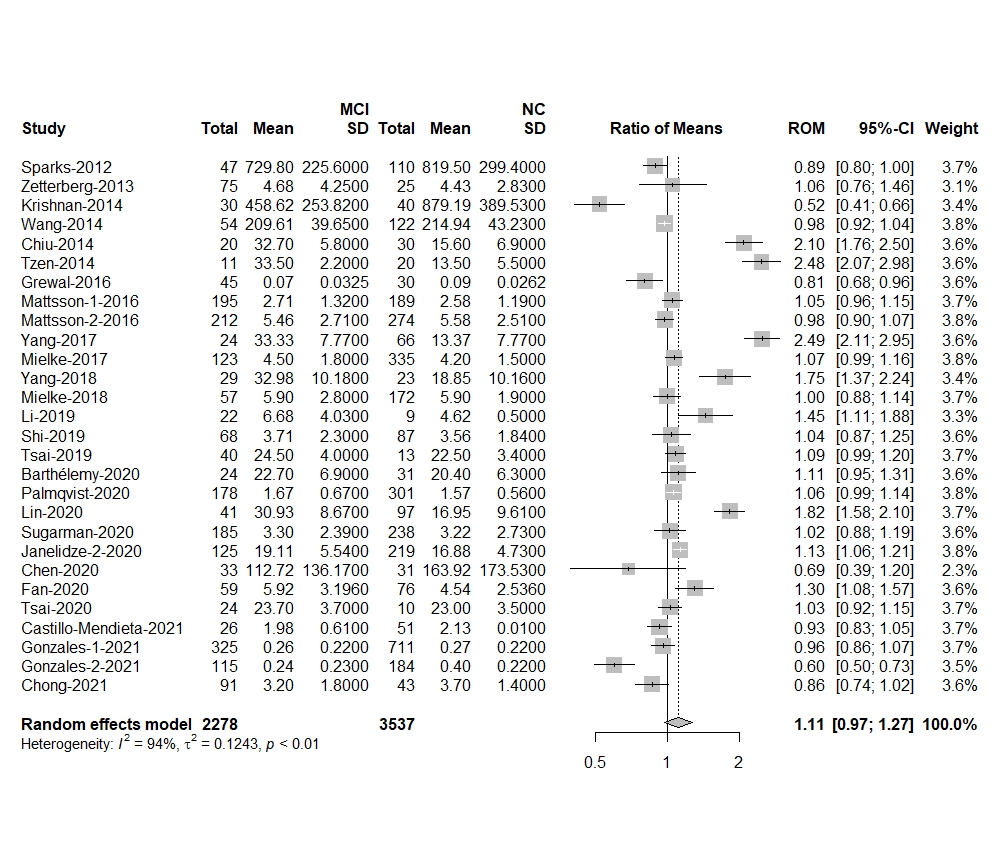


# Supplementary Fig.2. Subgroup Analysis:MCI to control ratio for blood p-tau


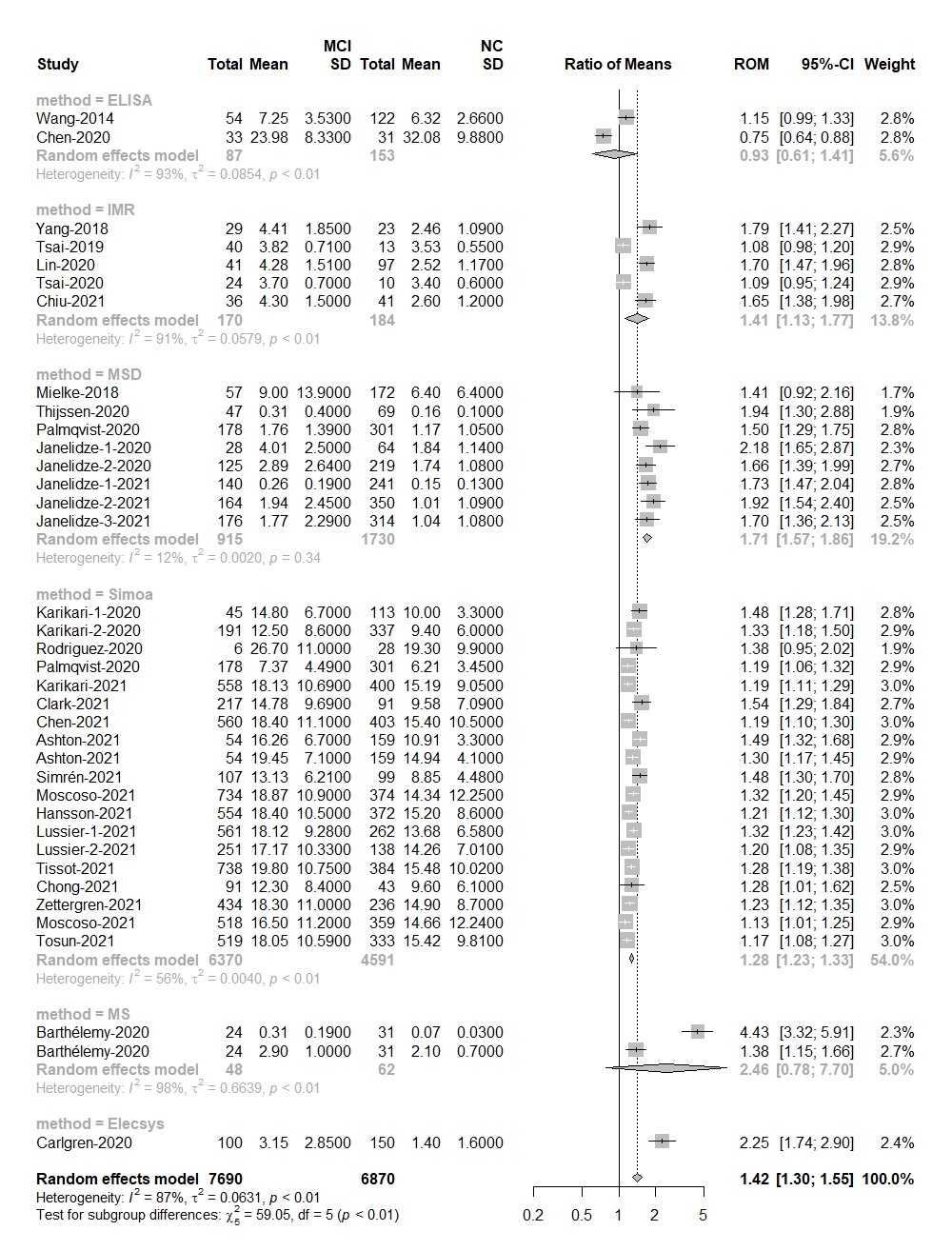


# Supplementary Fig.3. Subgroup Analysis:MCI to control ratio for blood t-tau


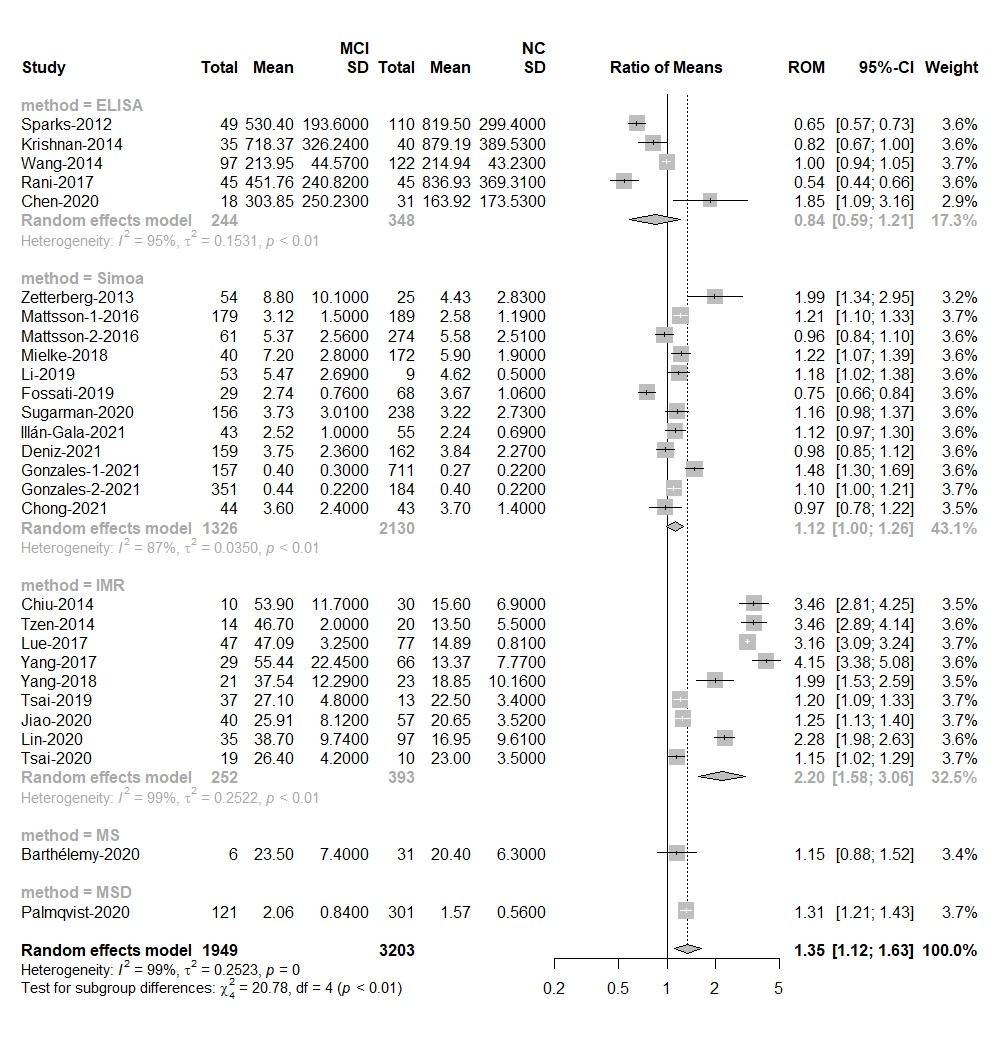


# Supplementary Fig.4. AD to control ratio for blood t-tau


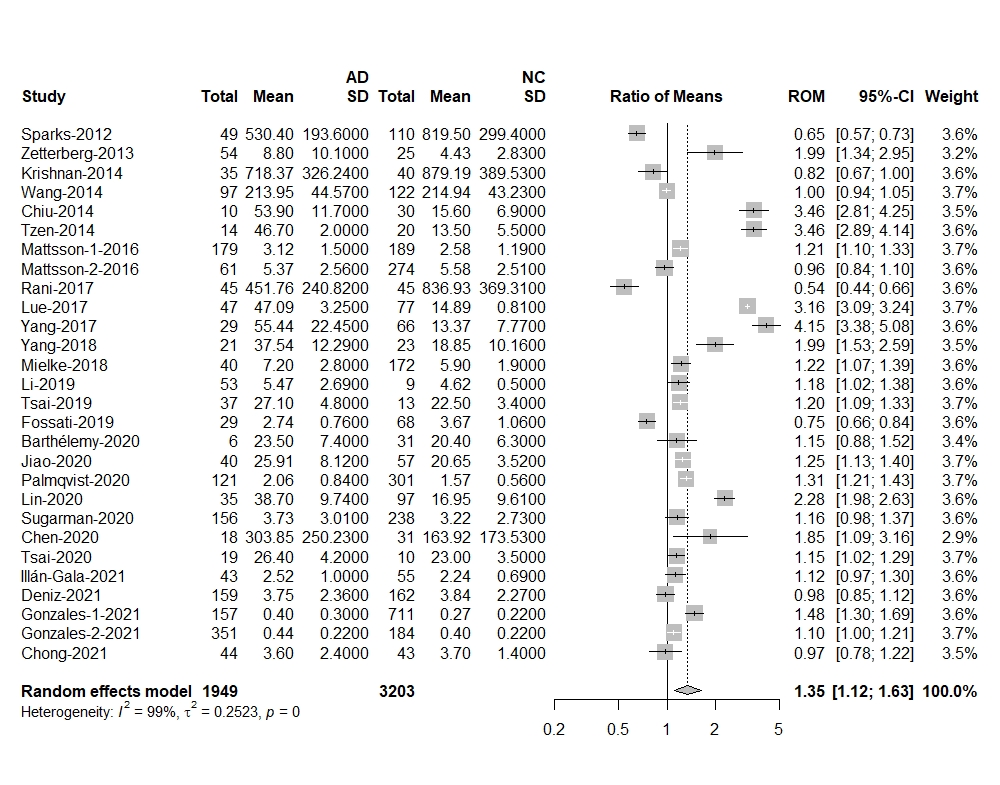


# Supplementary Fig.5. Subgroup Analysis: AD to control ratio for blood p-tau


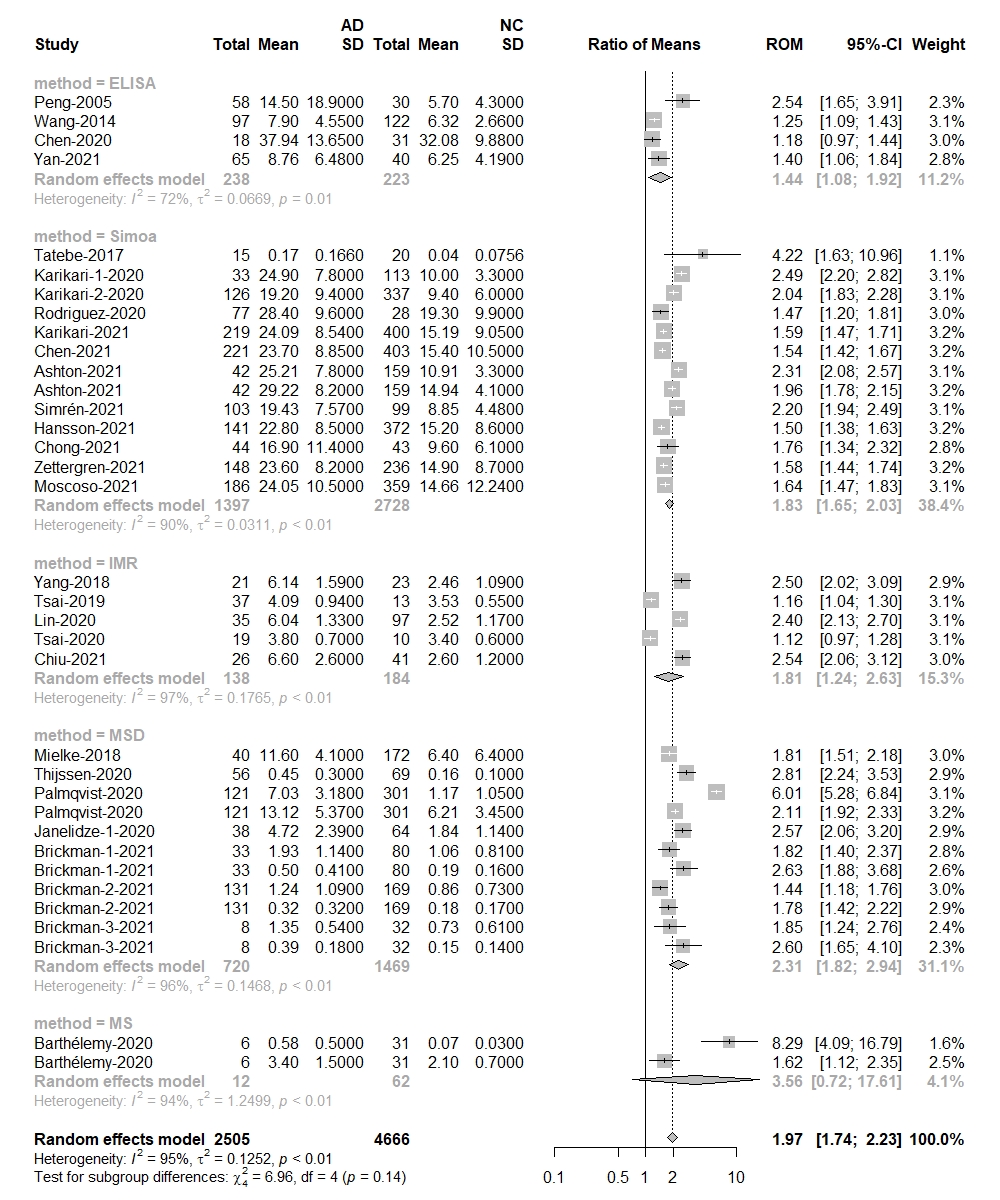


# Supplementary Fig.6. Subgroup Analysis:AD to control ratio for blood t-tau


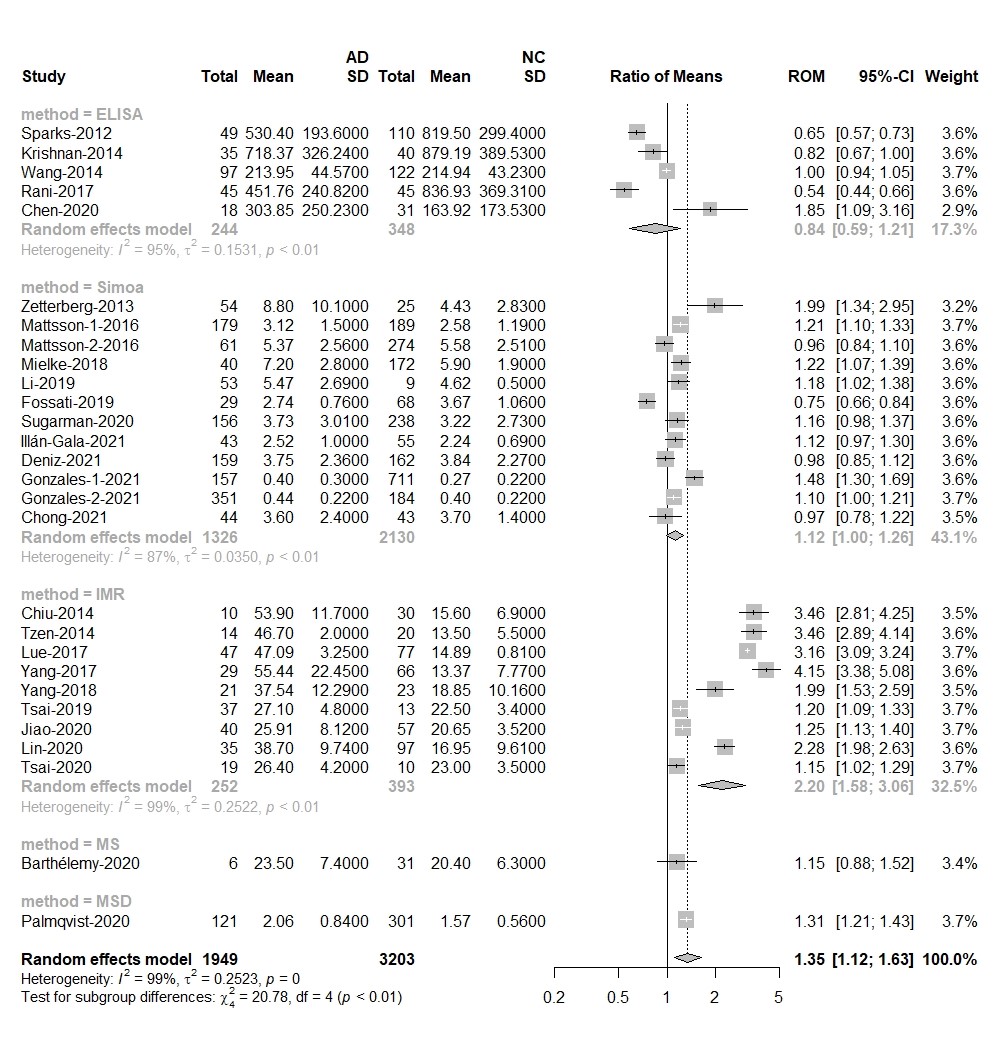


# Supplementary Fig.7. AD to MCI ratio for blood t-tau
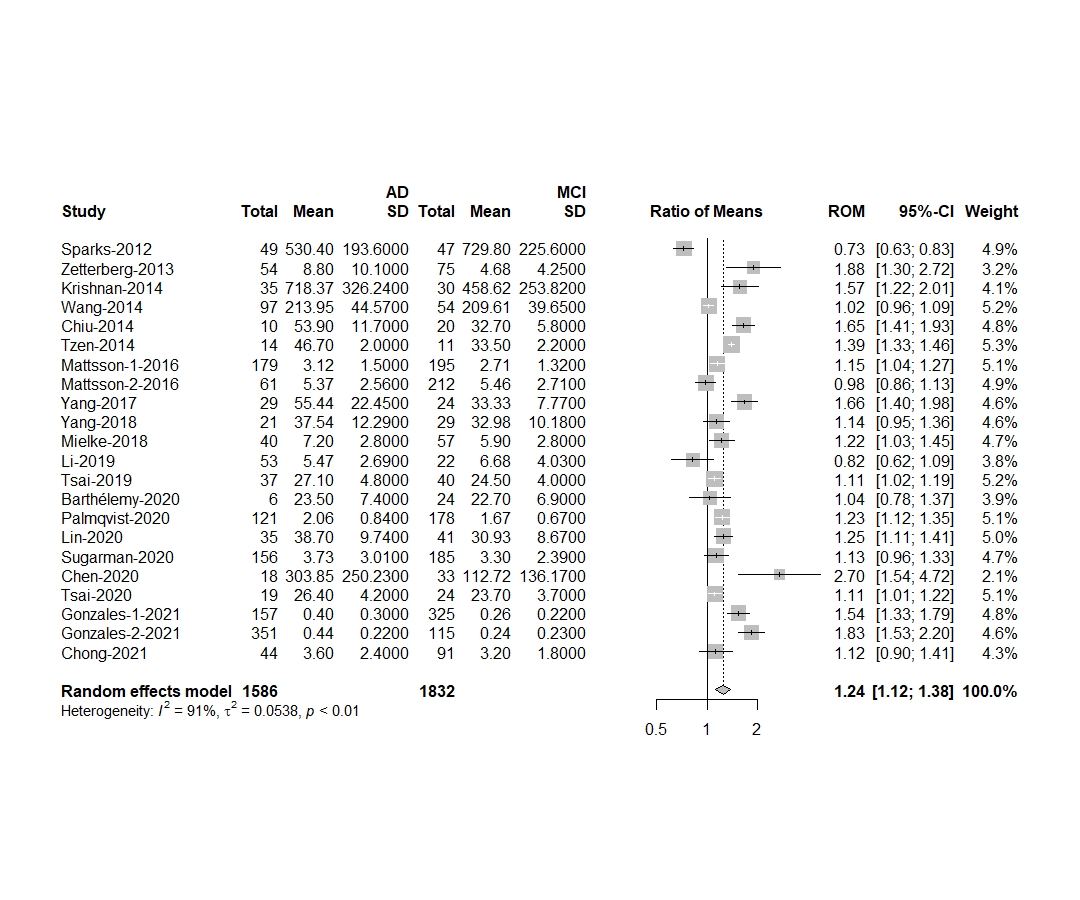


# Supplementary Fig.8. Subgroup Analysis:AD to MCI ratio for blood p-tau


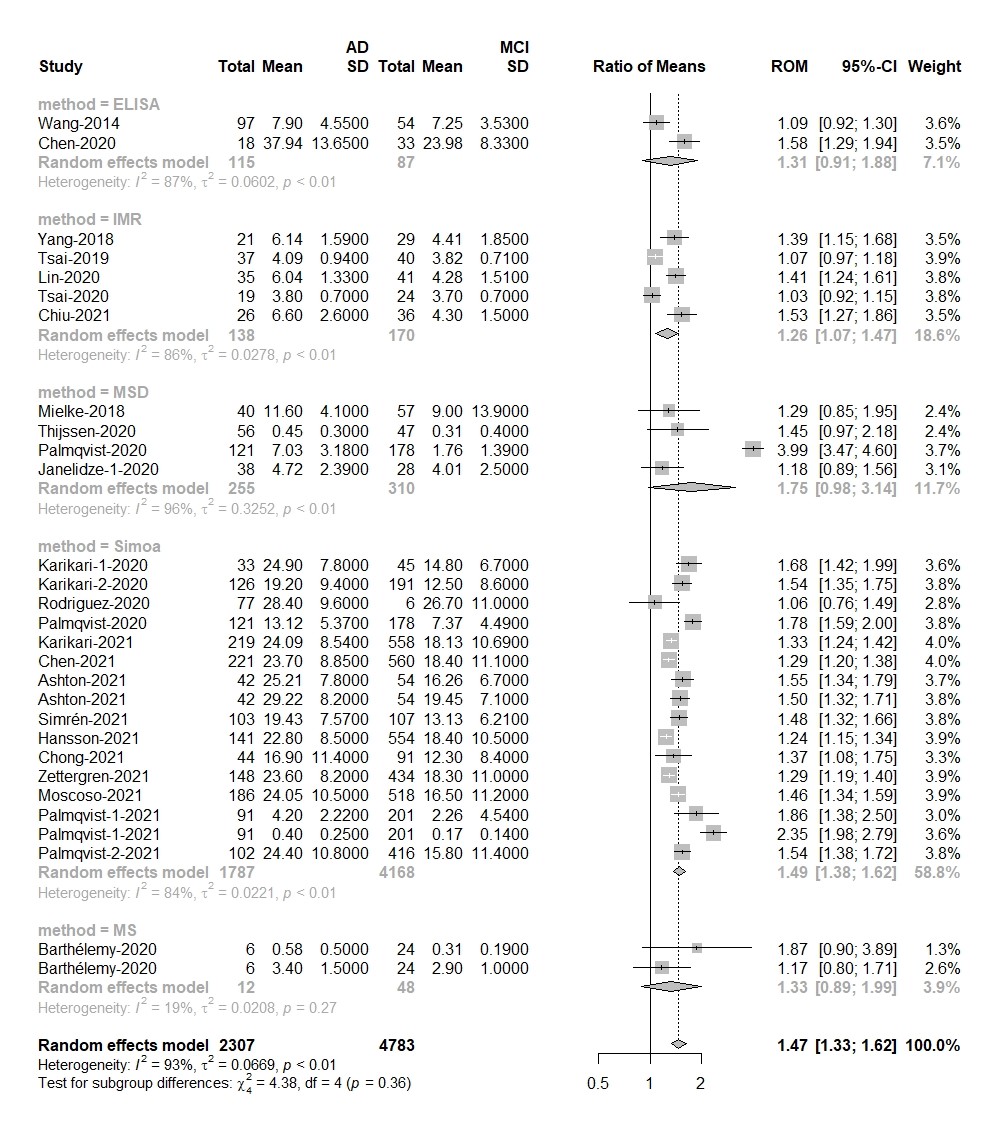


# Supplementary Fig.9. Subgroup Analysis:AD to MCI ratio for blood t-tau


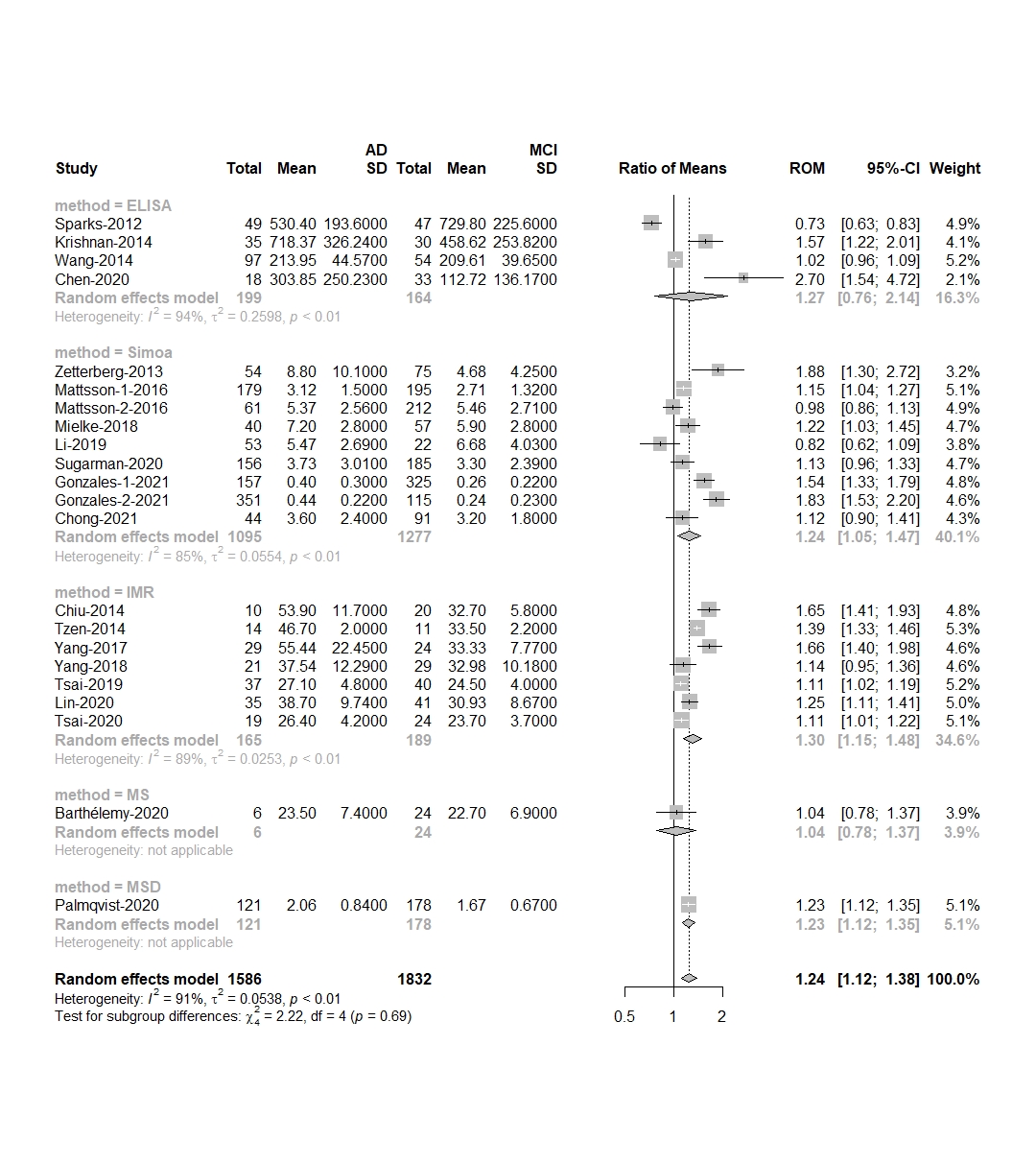


# Supplementary Fig.10. Funnel plot of blood p-tau in MCI samples vs controls


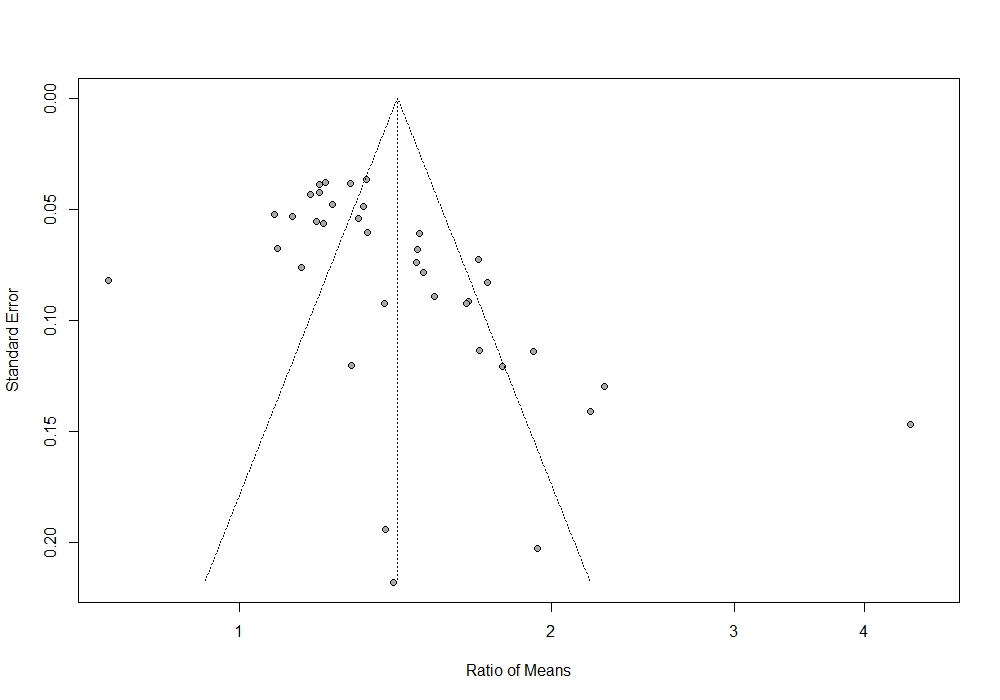


# Supplementary Fig.11. Funnel plot of blood t-tau in MCI samples vs controls


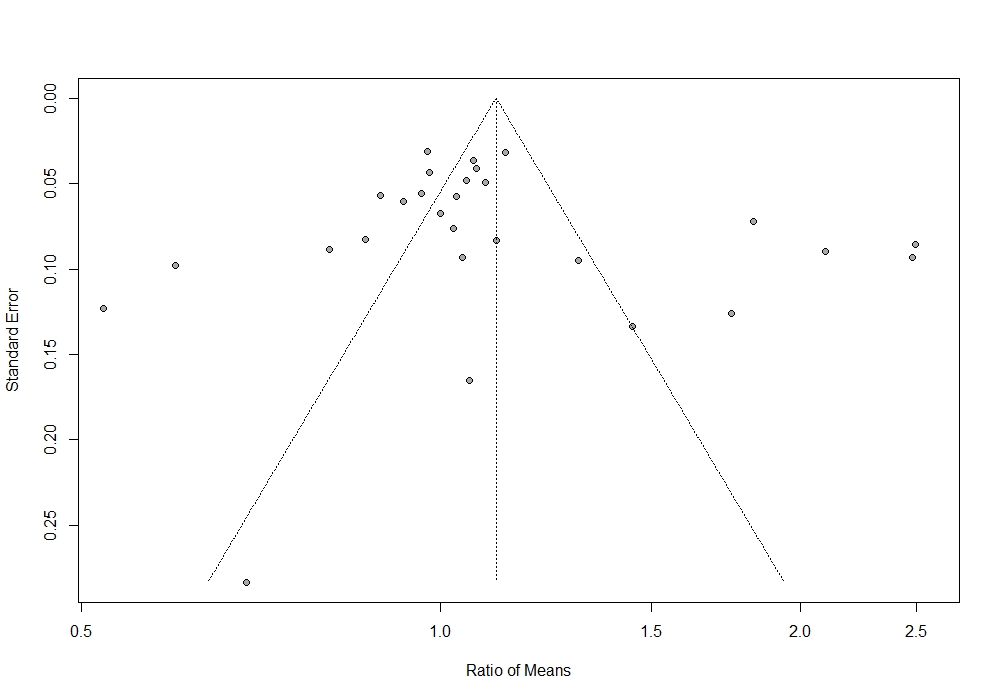


# Supplementary Fig.12. Funnel plot of blood p-tau in AD samples vs controls


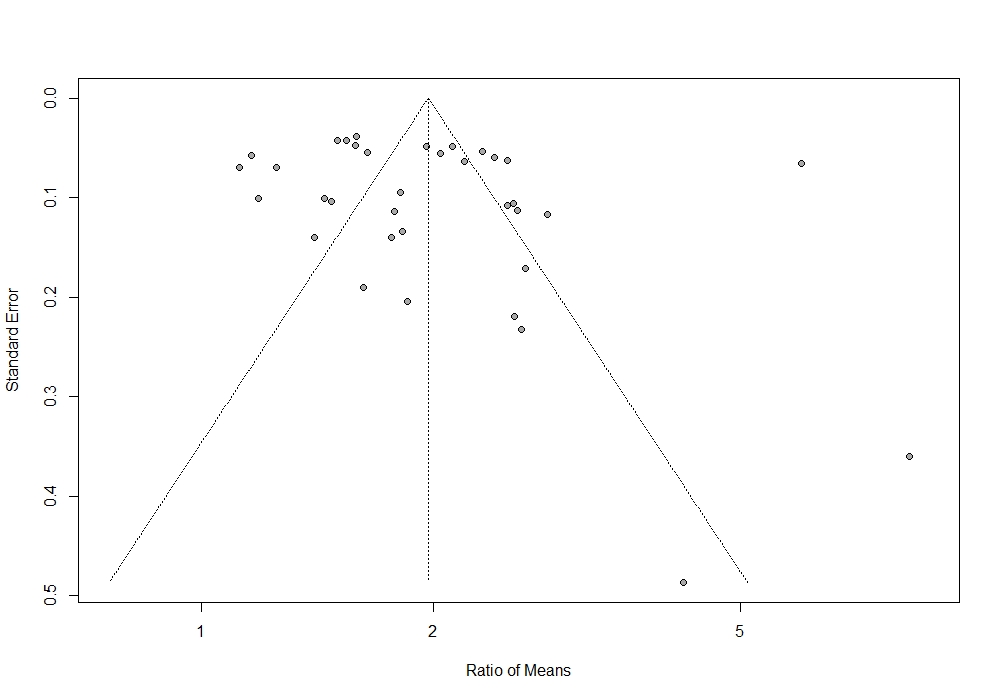


# Supplementary Fig.13. Funnel plot of blood t-tau in AD samples vs controls


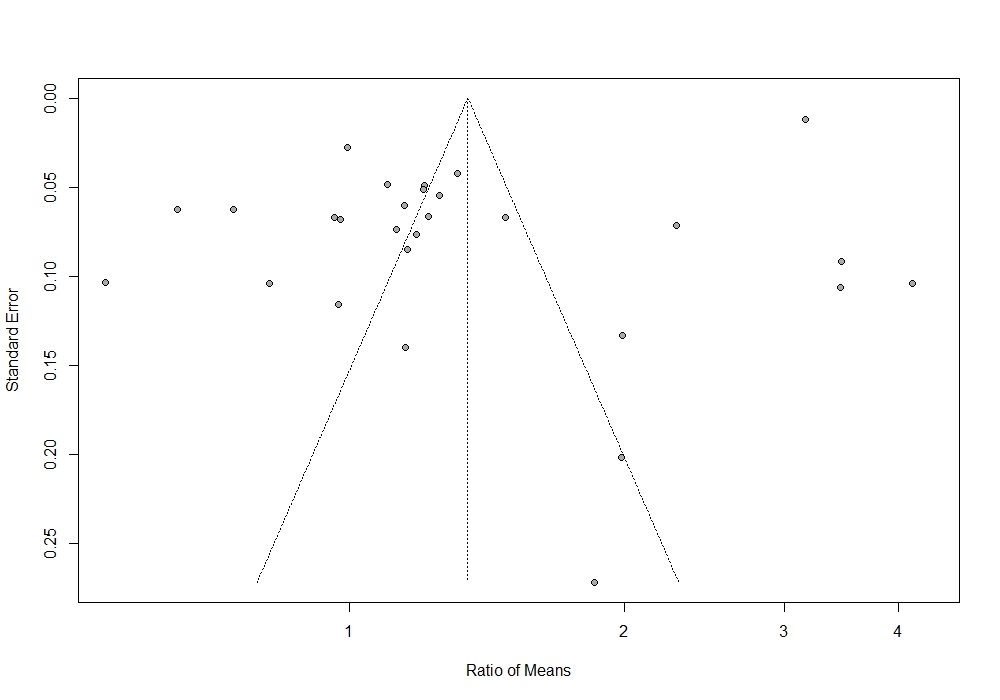


# Supplementary Fig.14. Funnel plot of blood p-tau in AD samples vs MCI


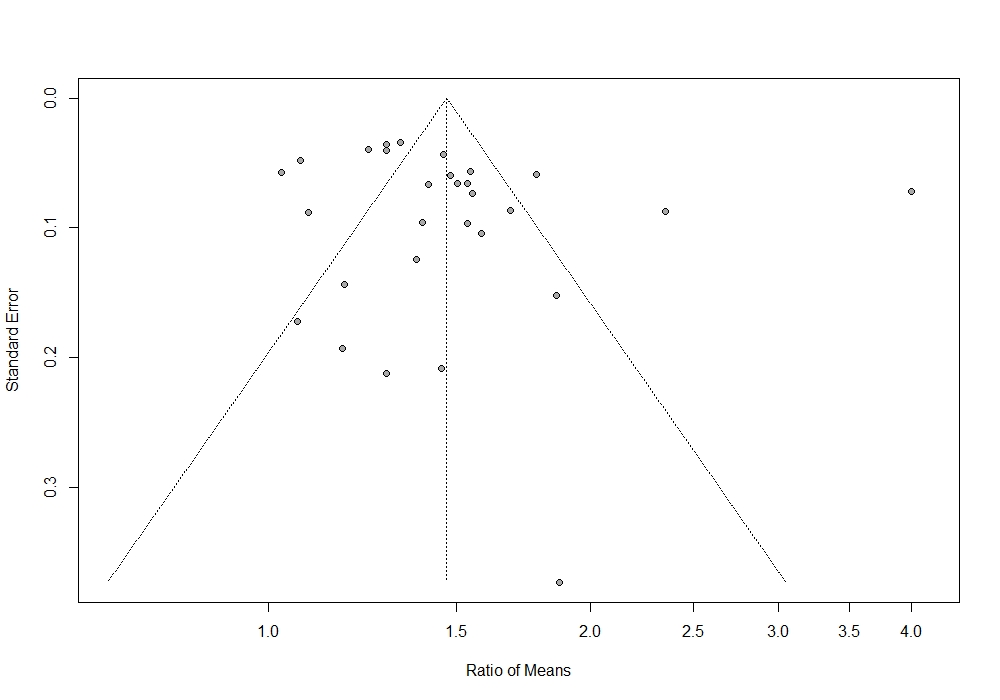


# Supplementary Fig.15. Funnel plot of blood t-tau in AD samples vs MCI


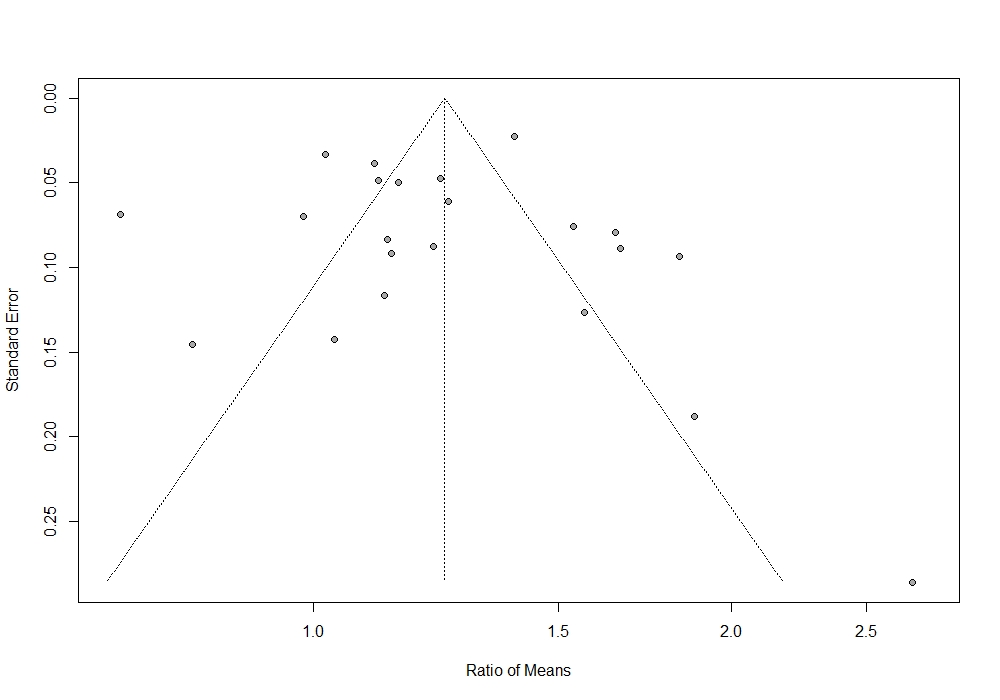


# Supplementary Fig.16. Sensitivity analysis of blood p-tau in MCI samples vs controls


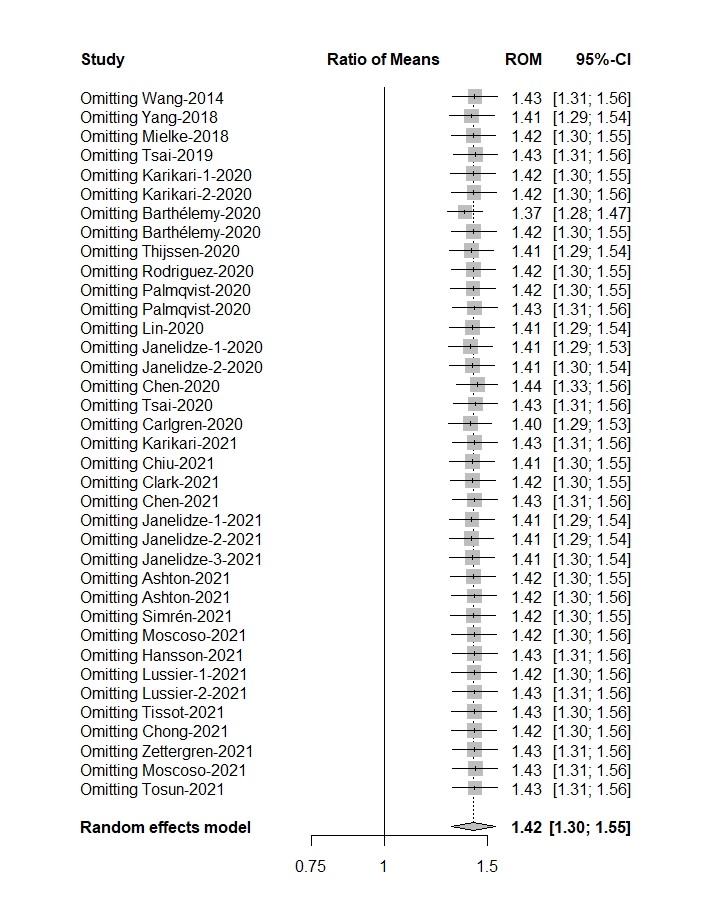


# Supplementary Fig.17. Sensitivity analysis of blood t-tau in MCI samples vs controls


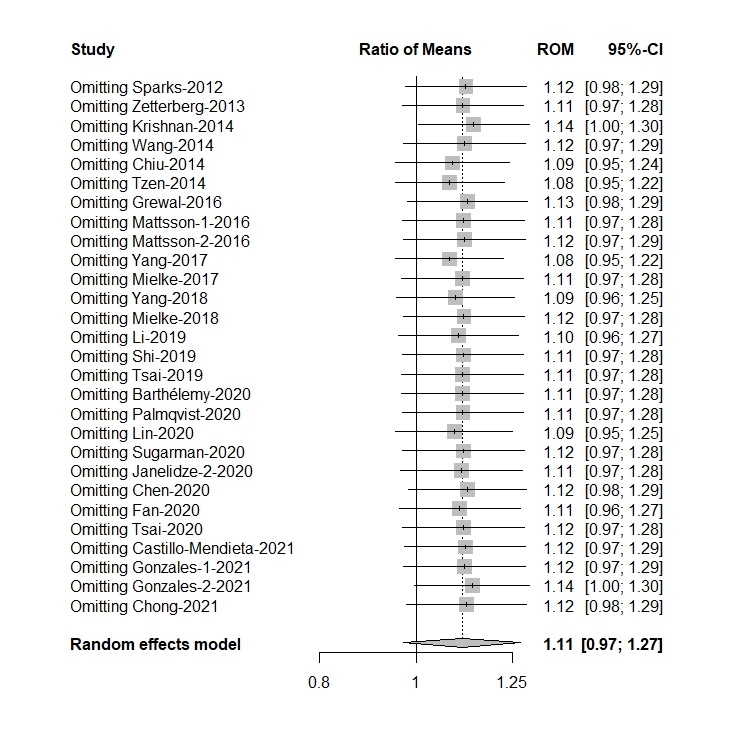


# Supplementary Fig.18. Sensitivity analysis of blood p-tau in AD samples vs controls


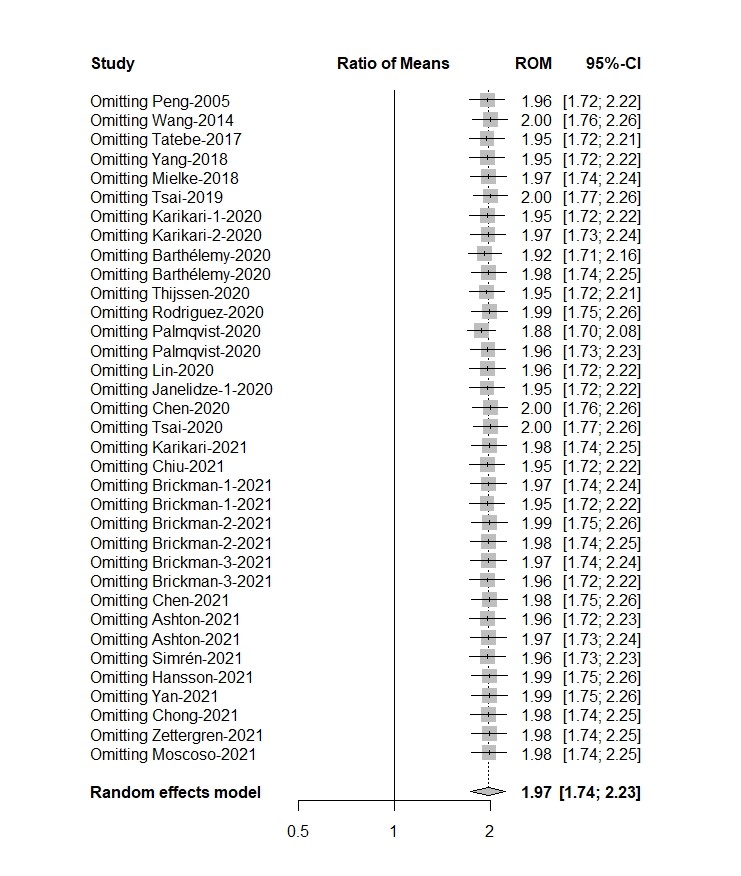


# Supplementary Fig.19. Sensitivity analysis of blood t-tau in AD samples vs controls


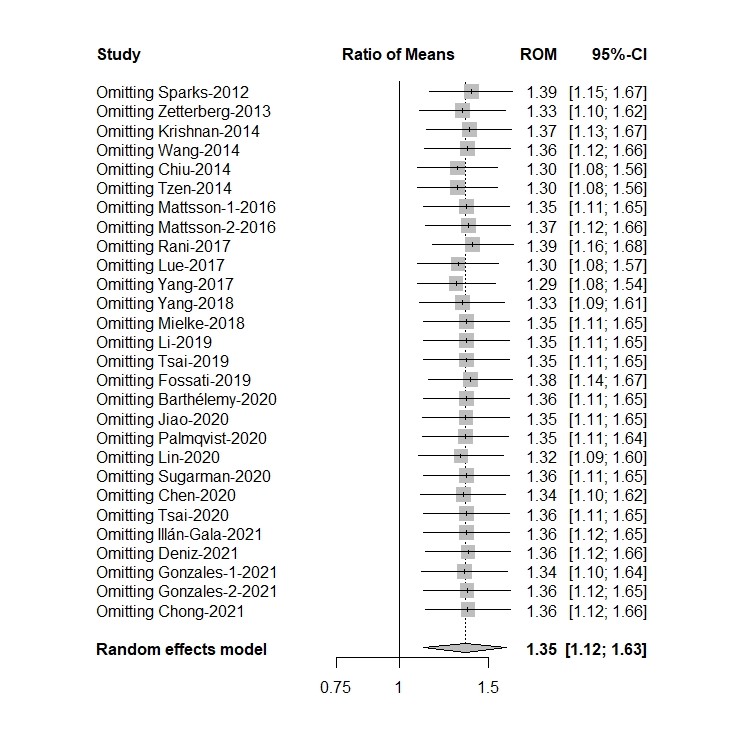


# Supplementary Fig.20. Sensitivity analysis of blood p-tau in AD samples vs MCI


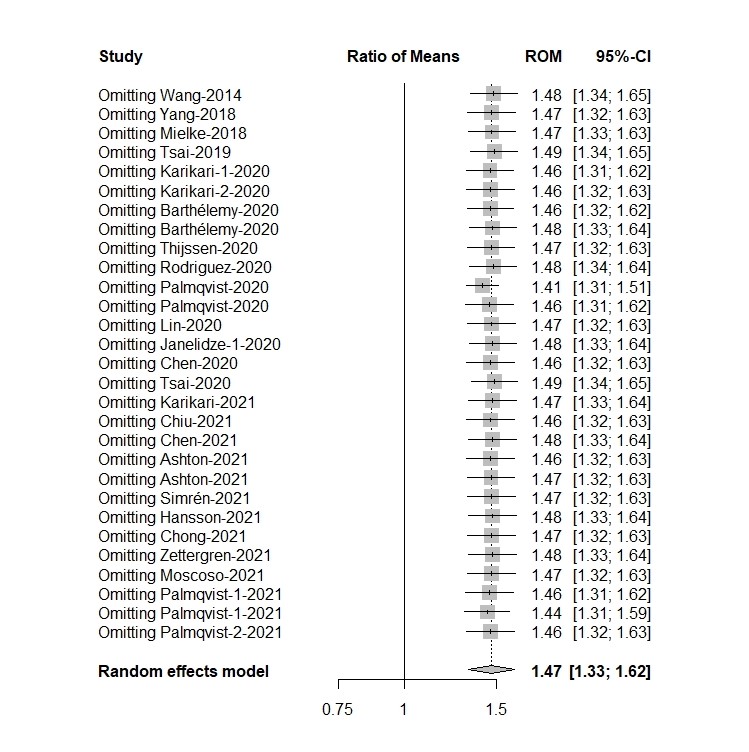


# Supplementary Fig.21. Sensitivity analysis of blood t-tau in AD samples vs MCI


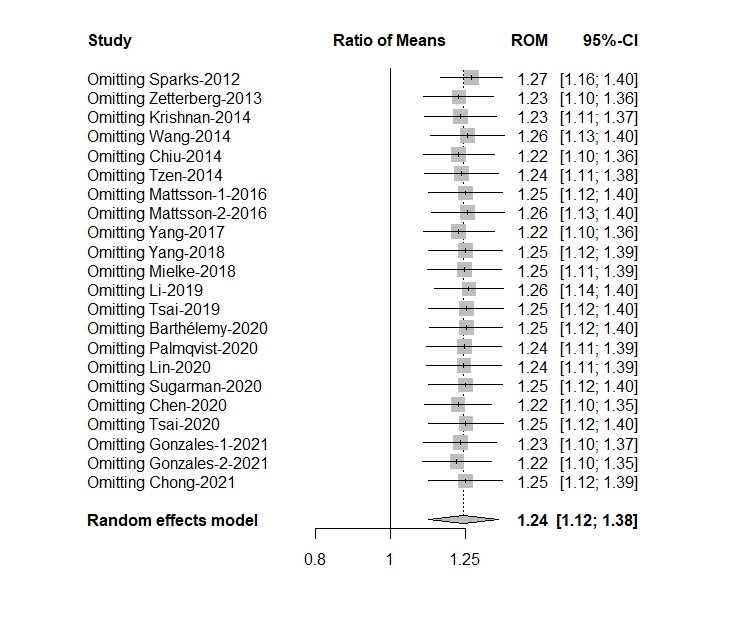

Supplement: Supplementary file 1 [file Data_Sheet_1.docx]
